# Comprehensive Characterization of Necroptosis-Related lncRNAs in Bladder Cancer Identifies a Novel Signature for Prognosis Prediction

**DOI:** 10.1155/2022/2360299

**Published:** 2022-06-06

**Authors:** Xiayu Kuang, Guogang Cai, Canxuan Li, Weibin Xie

**Affiliations:** ^1^Department of Urology, Shenshan Medical Center, Memorial Hospital of Sun Yat-sen University, Shanwei, Guangdong, China; ^2^Department of Urology, Sun Yat-sen Memorial Hospital, Sun Yat-sen University, Guangzhou, Guangdong, China; ^3^Guangdong Provincial Key Laboratory of Malignant Tumor Epigenetics and Gene Regulation, Sun Yat-sen Memorial Hospital, Sun Yat-sen University, Guangzhou, Guangdong, China; ^4^Guangdong Provincial Clinical Research Center for Urological Diseases, China

## Abstract

**Background:**

Bladder cancer (BC) is one of the most serious genitourinary malignant diseases with a poor prognosis. Necroptosis is a regulated form of cell death, and targeting necroptosis is emerging as a potential tumor therapy strategy. Nevertheless, the roles of necroptosis-related long noncoding RNAs (nrlncRNAs) in BC remains to be illustrated. This work is aimed at studying the clinical implications of nrlncRNAs in BC.

**Methods:**

The RNA-seq data and corresponding clinical data, downloaded from The Cancer Genome Atlas (TCGA) database, were utilized to obtain prognostic nrlncRNAs and construct a prediction nomogram for BC. The comprehensive profiling of the functional pathways, immune status, mutational landscape, and drug sensitivity related to the necroptosis-related lncRNA signature (NerRLsig) was performed.

**Results:**

Herein, a signature consisting of 12 necroptosis-related lncRNAs (AC015802.4, AL391807.1, AL078644.1, AC023825.2, AL132655.2, AP003352.1, STAG3L5P-PVRIG2P-PILRB, AC024451.4, MAP3K14-AS1, AL731567.1, AC010542.5, and AC009299.2) was constructed. The established signature can independently predict the poor overall survival of BC patients. Additionally, the NerRLsig had higher diagnostic validity compared to other clinicopathological variables, with a greater area under the receptor operating characteristic and concordance index curves. Finally, we found the differences in the functional signaling pathway, immune status, mutational profile, and drug sensitivity between the two subgroups.

**Conclusion:**

This research revealed that the prognostic NerRLsig and nomogram could accurately predict the prognosis of BC.

## 1. Introduction

Bladder cancer (BC), one of the most common urological neoplasms worldwide, is majorly nonmuscle-invasive with slow progression but a high recurrence rate [1, 2]. While muscle-invasive bladder cancer (MIBC) has worse outcomes and is more prone to develop metastatic disease [3, 4]. Despite the fact that immunotherapy using checkpoint inhibitors has revolutionized cancer care, the response rates remain unsatisfactory, with only a small percentage of patients responding to this treatment [5, 6]. Therefore, novel therapeutic strategies and individualized treatment plans for BC are needed.

Necroptosis is a regulated form of cell death characterized by cellular swelling and loss of cellular and organelle integrity [7, 8]. When tumor necrosis factor (TNF) binds to TNF receptor 1 (TNFR1) on the cell surface, RIPK1 (receptor-interacting serine/threonine kinase 1) is recruited to form a complex with RIPK3. This complex then phosphorylates MLKL (mixed lineage kinase domain-like pseudo kinase), leading to the execution of necroptosis [9, 10]. Several researchers found that the expression of hub mediators of the necrotic pathway is frequently downregulated in cancer cells, whereas high expression of necroptotic regulators indicates a favorable survival outcome for cancer patients. For instance, high RIPK3 expression predicted a favorable prognosis in colorectal cancer (CRC) [11], whereas low RIPK1 expression contributed to an unfavorable prognosis in hepatocellular carcinoma (HCC) [12]. Various chemotherapeutic agents or natural products have been shown to inhibit tumor progression by inducing necroptosis; for example, anthracycline and oxaliplatin-induced necroptosis cause tumors to display features of immunogenic cell death (ICD) and an activated anticancer immune response [13]. Pyruvate kinase M2 (PKM2) inhibitor shikonin-induced necroptosis can overcome the cisplatin resistance in BC [14]. These results suggest that triggering necroptosis in tumor cells may represent an attractive new strategy for cancer therapy.

In the human genome, only a very small number of protein-coding genes are present, and most of them are noncoding RNAs that cannot code for proteins, of which noncoding RNAs longer than 200 nucleotides are called lncRNAs [15, 16]. They mainly regulate gene expression by directly binding to microRNA [17, 18]. Abnormally expressed lncRNA has been confirmed to be involved in tumorigenesis and the development of BC, suggesting lncRNA has great potential to become therapeutic targets for BC [19, 20]. Nevertheless, these researches are generally limited to individual molecules. Recently, the role of lncRNA in the regulation of tumor necroptosis has been gradually discovered. For instance, Linc00176 could lead to necroptosis of HCC cells by targeting downstream factors, such as miR-9 and miR-185 [21]. lncRNAs could regulate necroptosis in cardiomyocytes via RIPK1/RIPK3 [22]. Moreover, lncRNA has been proven to help tumor cells evade immune destruction by limiting excessive inflammation, indicating its crucial role in the tumor microenvironment [23, 24]. However, the roles of necroptosis-related lncRNAs (nrlncRNAs) in BC are largely unknown.

Prognostic assessment is critical for monitoring follow-up and treatment decisions in BC patients. At present, a variety of staging and grading systems based on the clinicopathological characteristics of patients are recommended in clinical practice guidelines, which have certain guiding significance for the management and prognostic risk assessment of BC patients [25, 26]. However, these assessment methods still have limitations in the prognostic risk stratification of patients and cannot well distinguish the prognostic risk of patients with the same clinical stage or similar histological grades [27]. Several studies have reported the role of prognostic gene models based on genomics data in the prognosis prediction of BC patients [28–30]. Therefore, we created a prognostic necroptosis-related lncRNA signature (NerRLsig) for BC and investigated its potential ability in assessing the immune landscape and mutational status and predicting the effects of chemotherapy and targeted therapies.

## 2. Materials and Methods

### 2.1. Data Acquisition

The RNA-seq data containing 406 bladder cancer specimens and 19 normal controls, were acquired from the TCGA portal (https://tcga-data.nci.nih.gov/tcga/) [31]. Correlative clinicopathological data and somatic mutation data for BC patients were also obtained from TCGA Bladder Cancer databases. Next, a list of 50 necroptosis-related genes (NRGs) was collated from the literature and Molecular Signatures Database (M24779.gmt gene set). Drug sensitivity data of chemotherapy or targeted drugs were available on the Genomics of Drug Sensitivity in Cancer (GDSC) (http://www.cancerrxgene.org/) [32] database and CellMiner database (https://discover.nci.nih.gov/cellminer) [33]

### 2.2. Study Design

First, Pearson's correlation analysis between lncRNAs and NRGs was performed and candidate nrlncRNAs were identified when the correlation coefficient > 0.4 and *p* value < 0.05. Then, the differentially expressed nrlncRNAs (DEnrlncRNAs) between tumor and adjacent nontumorous specimens were screened via the “limma” R package; log2 |fold change| > 1 and false discovery rate (FDR) < 0.05 were set as the cut-off value. Next, 406 cases with survival data were randomly divided into two cohorts, a training cohort and a testing cohort, in a ratio of 7 : 3. The univariate Cox regression analysis was applied to identify prognosis-related DEnrlncRNAs in the training cohort (*p* < 0.01). The least absolute shrinkage and selection operator (LASSO) Cox regression analysis were carried out to determine hub DEnrlncRNAs and construct a prognostic NerRLsig. The risk score was computed according to the following formula: risk score = sum (expression for each lncRNA∗coefficient for each lncRNA). Patients in the training cohort were separated into the low-NerRLsig and high-NerRLsig subgroups according to the median risk score of the training set. Kaplan-Meier survival curves were employed to compare the different OS between two subgroups using the “survminer” R package. Receiver operating characteristic (ROC) curves were conducted to estimate the predictive capability of the NerRLsig using the “survivalROC” R package. Risk scores for patients in the test and entire cohorts were calculated using the formula obtained from the training set, and patients were classified into the high-NerRLsig and low-NerRLsig subgroups based on the median risk values in the training set, and the prognostic value of the NerRLsig in the training and total sample sets was tested by Kaplan-Meier survival curves and ROC curves. Univariate and multivariate Cox regression analyses were performed in the entire cohort to test the independence of the NerRLsig in predicting the overall survival prognosis of patients with BC. Next, a nomogram for predicting patient OS was built and calibration plots were created to valuated the performance of the nomogram using the “rms” R package. The ROC curves of the predictive nomogram were also drawn using “survivalROC” R package. Furthermore, conformance indexes and ROC curves were employed to explore whether the prediction accuracy of the NerRLsig has advantages over traditional pathological variables. Additionally, the chi-square test and the Wilcoxon rank-sum test were utilized to study the correlation between NerRLsig and clinical features.

### 2.3. Function Analysis of the NerRLsig

The differentially expressed genes (DEGs) between the two subgroups were screened using the “edgeR” R package; log2 |fold change| > 1 and false discovery rate (FDR) < 0.05 set as the cut-off value. Gene ontology (GO) and Kyoto Encyclopedia of Genes and Genomes (KEGG) analyses were conducted to explore potential molecular and biological mechanisms related to the NerRLsig, with *p* < 0.05 indicating enrichment for significant functional annotation. To further explore the mechanism of action, the gene set enrichment analysis (GSEA) was performed to investigate the NerRLsig-related signaling pathways, with *p* < 0.05 indicating enrichment for significant functional annotation.

### 2.4. Immune Landscape Assessment and Mutation Analysis of the NerRLsig

To systematically describe the link between the immune microenvironment and prognostic signature, we calculated the relative abundances of 22 tumor-infiltrating immune cells using the CIBERSORT algorithm, quantified 13 immune-associated signaling pathways using the single-sample enrichment analysis (ssGSEA), and then compared the differences in immune cells and immune-related pathways between subgroups via the Wilcoxon signed-rank test. In addition, transcriptomic data for common immune checkpoint inhibitors were extracted from the TCGA bladder cancer dataset, and differences in immune checkpoint expression levels between the two groups were compared using the Wilcoxon signed-rank test. Additionally, we created a waterfall plot using “maftools” R package to initially explore the mutational status of patients in the high-NerRLsig and low-NerRLsig groups.

### 2.5. Sensitivity to Chemotherapy Drugs of the NerRLsig

To estimate the sensitivity of high-NerRLsig and low-NerRLsig subgroups to different chemotherapeutics, we calculated the half-maximal inhibitory concentrations (IC50s) of common chemotherapeutics using the “pRRophetic” R package and compared the differences in IC50 values between subgroups using the Wilcoxon signed-rank test. Additionally, Pearson's correlation analysis was performed to determine the correlation between hub nrlncRNAs and drug sensitivity.

### 2.6. Statistical Analysis

All statistical analyses were performed using R 3.6.1 software. Statistical significance was considered to be a *p* < 0.05 if not otherwise stated.

## 3. Results

### 3.1. Identification of Prognostic DEnrlncRNAs in BC

**Figure 1:**
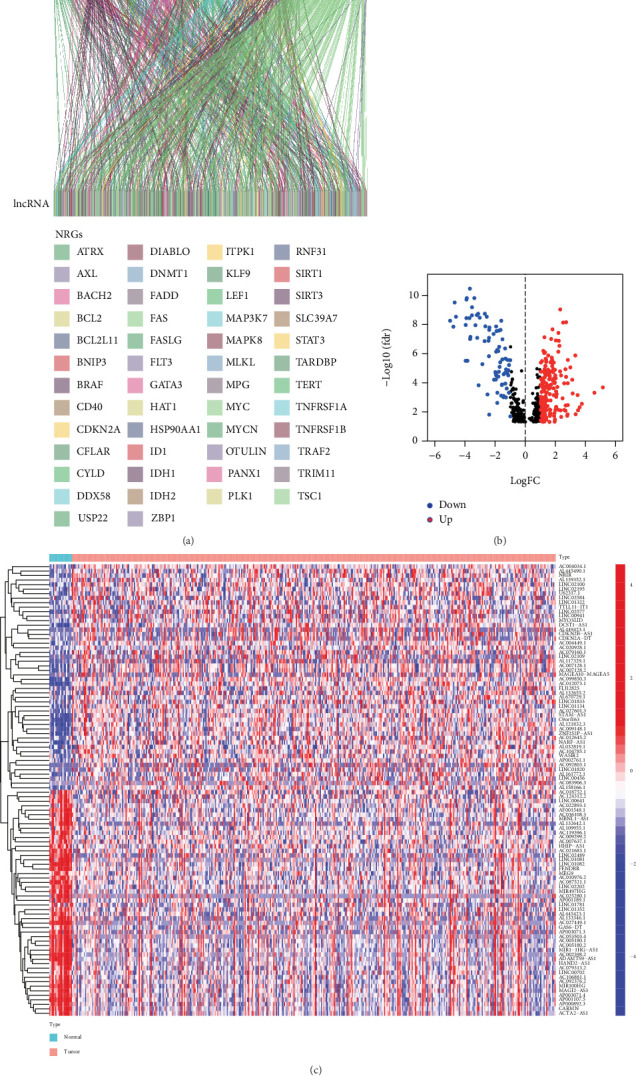
Selection of necroptosis-related lncRNAs in bladder cancer patients. (a) Sankey diagram for the network of necroptosis-related genes and lncRNAs. (b) Volcano plot for the differentially expressed necroptosis-related lncRNAs. (c) Heatmap for the differentially expressed necroptosis-related lncRNAs.

**Figure 2:**
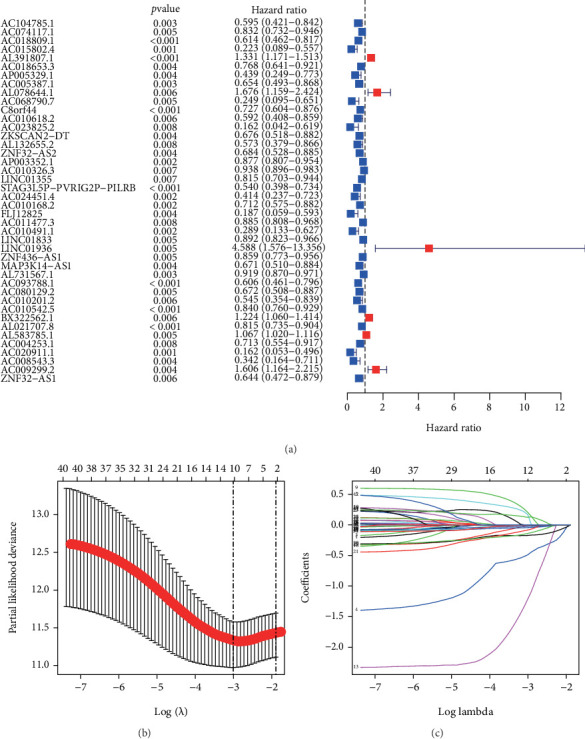
Construction a prognostic necroptosis-related lncRNA signature (NerRLsig) in the TCGA training set. (a) 42 differentially expressed necroptosis-related lncRNAs were identified by univariate Cox analysis. (b) LASSO Cox regression. (c) Determination of the optimal LASSO settings.

Herein, we obtained 504 nrlncRNAs via Pearson's coexpression analysis (Figure 1(a) and Supplementary Table 1), of which 278 were differentially expressed between the tumor group and normal groups (Supplementary Table 2). The expression profiles of DEnrlncRNAs were visualized as volcano and heatmaps (Figures 1(b) and 1(c)). These 278 DEnrlncRNAs were used as candidate markers for subsequent prognostic model construction. Then, 42 prognostic DEnrlncRNAs, including AC104785.1, AC074117.1, AC018809.1, AC015802.4, AL391807.1, AC018653.3, AP005329.1, AC005387.1, AL078644.1, AC068790.7, C8orf44, AC010618.2, AC023825.2, ZKSCAN2-DT, AL132655.2, ZNF32-AS2, AP003352.1, AC010326.3, LINC01355, STAG3L5P-PVRIG2P-PILRB, AC024451.4, AC010168.2, FLJ12825, AC011477.3, AC010491.1, LINC01833, LINC01936, ZNF436-AS1, MAP3K14-AS1, AL731567.1, AC093788.1, AC080129.2, AC010201.2, AC010542.5, BX322562.1, AL021707.8, AL583785.1, AC004253.1, AC020911.1, AC008543.3, AC009299.2, and ZNF32-AS1, were identified based on univariate Cox regression analysis (*p* < 0.01) (Figure 2(a) and Supplementary Table 3), which were enrolled in LASSO regression analysis and 12 of them were outperformed in the constructing the NerRLsig, including AC015802.4, AL391807.1, AL078644.1, AC023825.2, AL132655.2, AP003352.1, STAG3L5P-PVRIG2P-PILRB, AC024451.4, MAP3K14-AS1, AL731567.1, AC010542.5, and AC009299.2 (Figures 2(b) and 2(c) and Supplementary Table 4). The risk score formula was as follows: (expression level of AC015802.4∗(−0.488) + expression level of AL391807.1∗(0.166) + expression level of AL078644.1∗(0.204) + expression level of AC023825.2∗(−1.212) + expression level of AL132655.2∗(−0.005) + expression level of AP003352.1∗(−0.045) + expression level of STAG3L5P − PVRIG2P − PILRB∗(−0.172) + expression level of AC024451.4∗(−0.084) + expression level of MAP3K14 − AS1∗(−0.140) + expression level of AL731567.1∗(0.025) + expression level of AC010542.5∗(−0.003) + expression level of AC009299.2∗(0.123)). Based on the median risk score, BC patients were separated into the high-NerRLsig and low-NerRLsig subgroups.

### 3.2. Assessment and Validation of the NerRLsig

**Figure 3:**
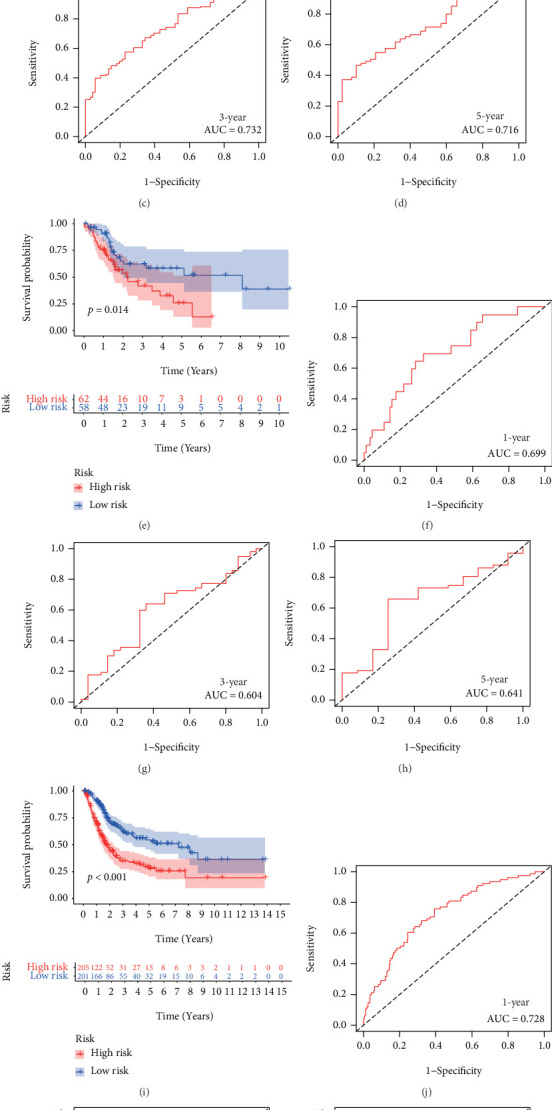
Assessment and validation of the prognostic necroptosis-related lncRNA signature (NerRLsig). (a, e, i) The KM survival curves of the low-risk group and the high-risk group in the TCGA training set, testing set, and entire set, respectively. (b–d) ROC curves were used to assess the efficiency of the risk signature for predicting 1-year, 3-year, and 5-year survival rates in the TCGA training set. (f–h) ROC curves were used to assess the efficiency of the risk signature for predicting 1-year, 3-year, and 5-year survival rates in the TCGA testing set. (j–l) ROC curves were used to assess the efficiency of the risk signature for predicting 1-year, 3-year, and 5-year survival rates in the TCGA entire cohort.

**Figure 4:**
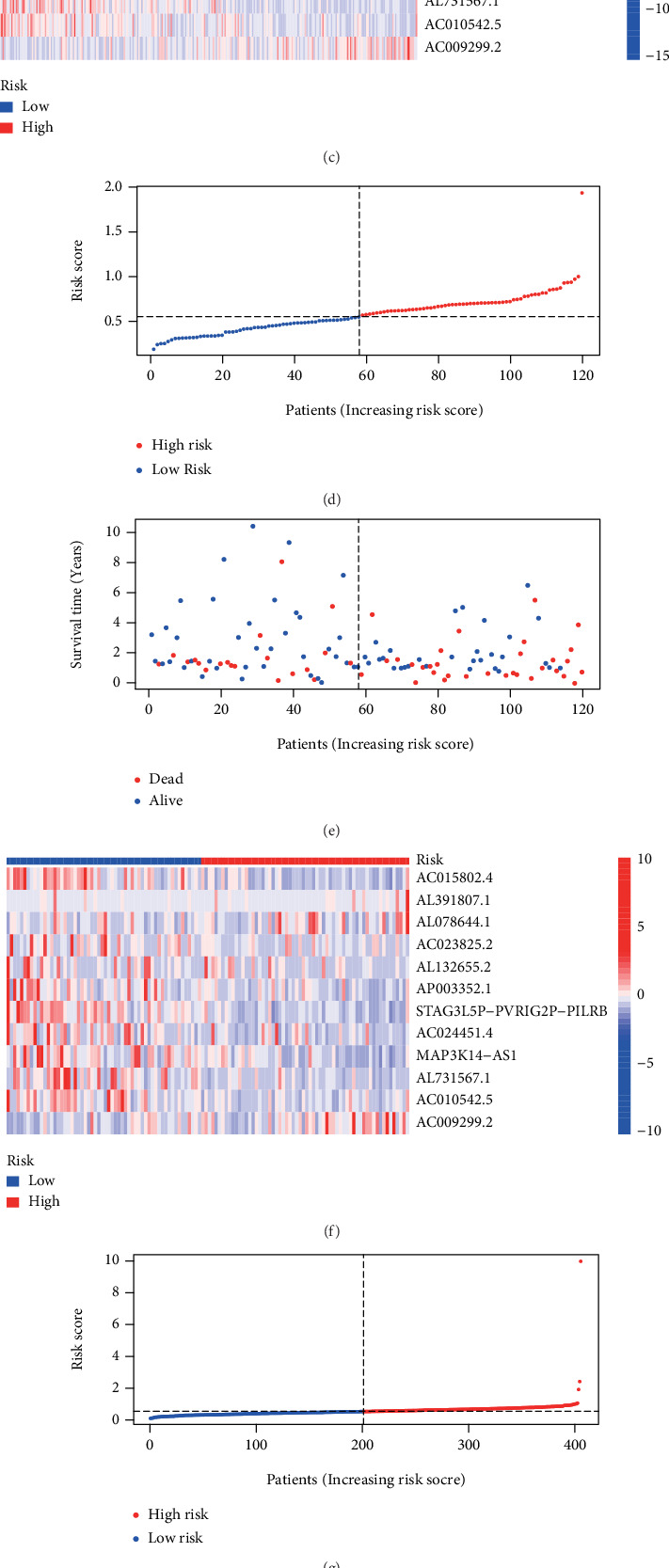
Distribution of bladder cancer (BC) patients based on the risk score. (a, d, g) Risk curve and (b, e, h) scatter plot for the risk score and survival status of each BC case. (c, f, i) Heatmap showing the expression profiles of necroptosis-related lncRNAs in the high-risk group and the low-risk group.

In the training set, Kaplan-Meier survival analysis revealed that the low-NerRLsig group had a greater OS than the high-NerRLsig group (*p* < 0.001) (Figure 3(a)). The same survival outcomes were both observed in the test set (*p* = 0.001) and the entire set (*p* < 0.001) (Figures 3(e) and 3(i)). Time-dependent ROC analysis showed that the AUC of the risk score predicted OS was 0.743 at 1 year, 0.732 at 3 years, and 0.716 at 5 years in the training cohort (Figures 3(b)–3(d)); 0.699 at 1 year, 0604 at 3 years, and 0.641 at 5 years in the testing dataset (Figures 3(f)–3(h)): and 0.728 at 1 year, 0.698 at 3 years, and 0.698 at 5 years in the entire cohort (Figures 3(j)–3(l)). Figures 4(a)–4(i) show the distribution of the risk score, survival status, and the expression heatmap of nrlncRNAs in the training, testing, and entire cohorts.

### 3.3. Clinical Implications of the NerRLsig

**Figure 5:**
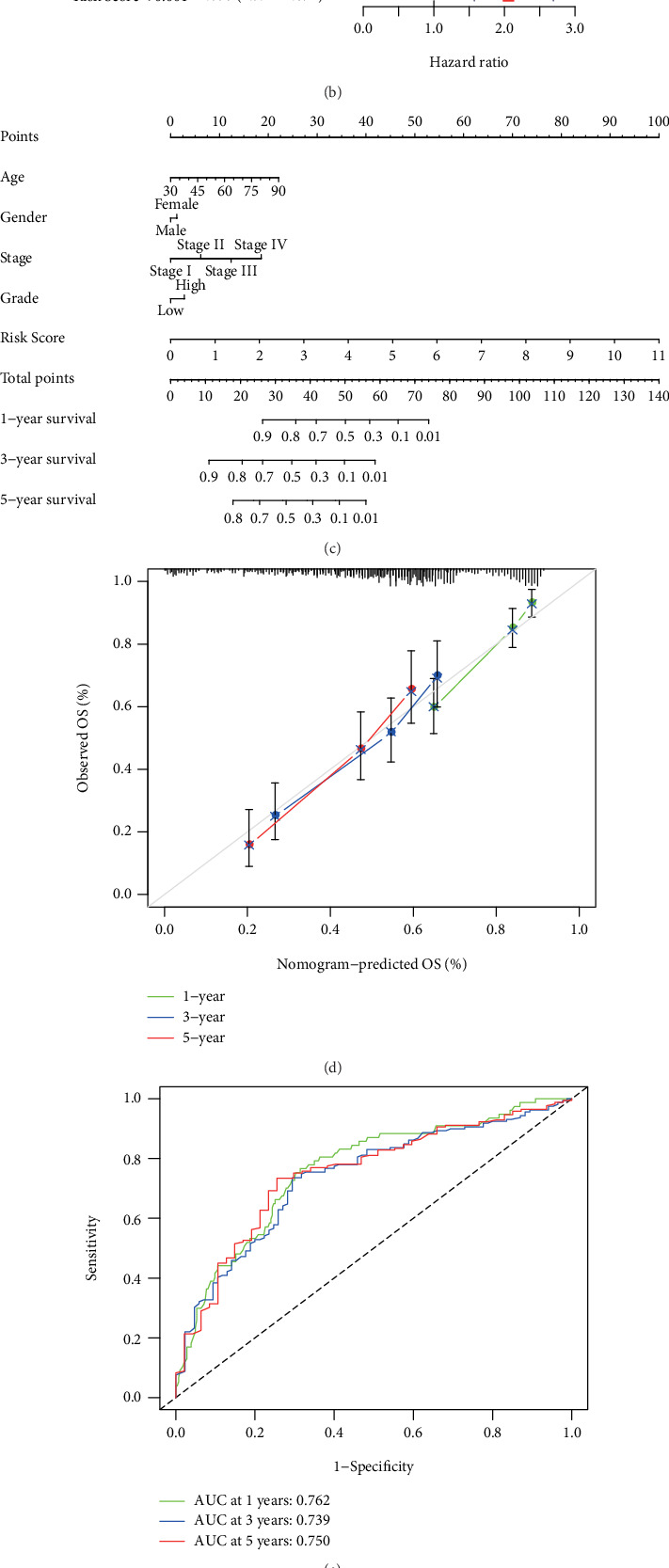
Prognostic value of the necroptosis-related lncRNA signature (NerRLsig) in the entire cohort. (a, b) Univariate and multivariate Cox regression analyses for the NerRLsig as an independent prognostic factor. (c) A prognostic nomogram for the NerRLsig and other clinicopathological factors. (d) 1-year, 3-year, and 5-year survival rate calibration curves of the line chart. (e) ROC curves of 1-, 3-, and 5-year survival. (f) Concordance indexes of both clinical features and risk score. (g) ROC curves of both clinical features and risk score.

**Figure 6:**
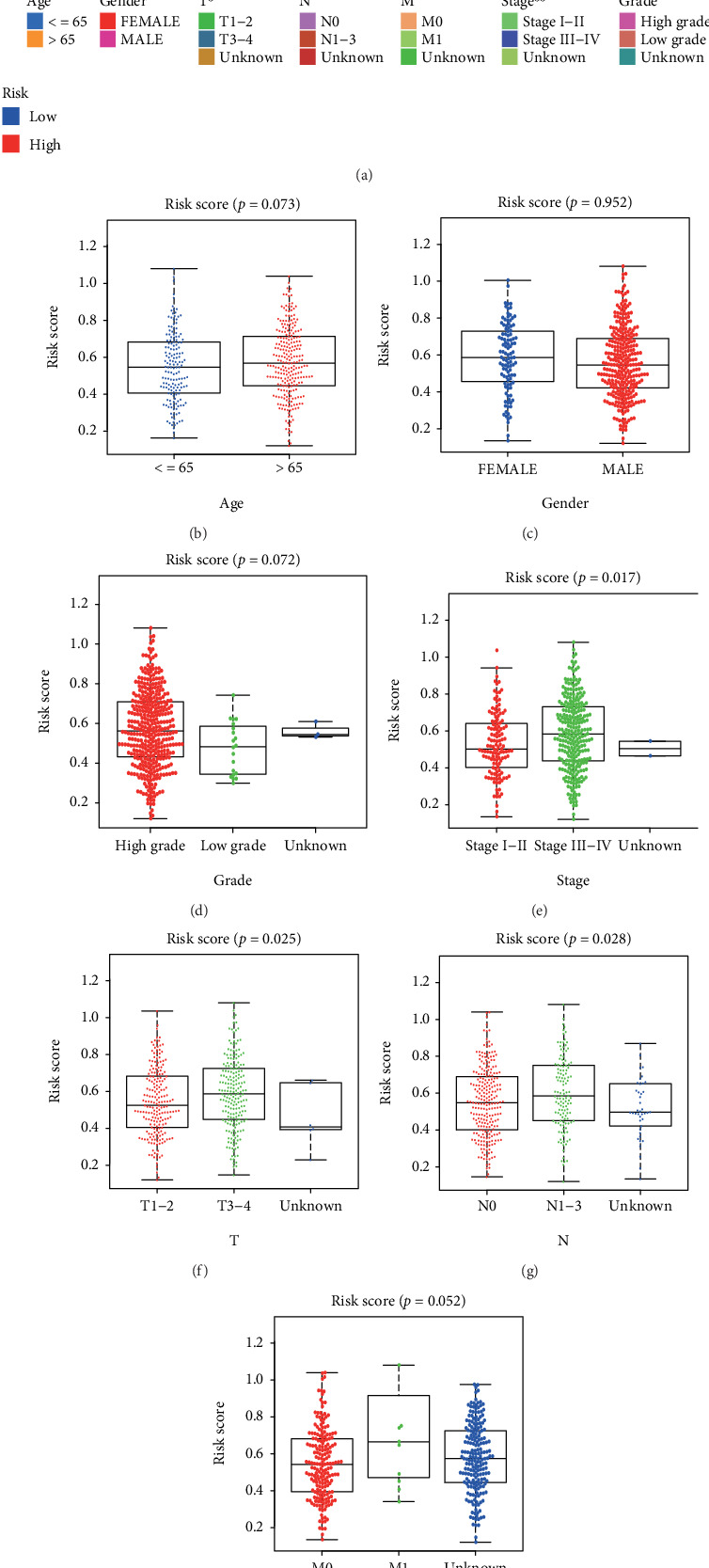
The risk score correlated with clinicopathological features in bladder cancer. (a) Heatmap revealed a significant pT stage and clinical stage between high-risk and low-risk groups. (b–g) The risk score in different age (b), gender (c), tumor grade (d), clinical stage (e), pT stage (f), pN stage (g), and pM stage (h) of bladder cancer patients. ⁣^∗^*p* < 0.05 and^∗∗^*p* < 0.01.

Firstly, we confirmed that NerRLsig was an independent prognostic factor for BC (*p* < 0.01) (Figures 5(a) and 5(b) and Supplementary Table 5). Second, a predictive nomogram was created using NerRLsig and clinicopathological characteristics to predict 1-, 3-, and 5-year OS in BC patients (Figure 5(c)). The calibration curves demonstrated that the prognostic nomogram was robust and precise (Figure 5(d)). Thirdly, the AUCs of the nomogram in the ROC curves were 0.762, 0.739, and 0.778 at 1, 3, and 5 years, respectively (Figure 5(e)). Additionally, as shown in Figure 5(f), the prognostic signature concordance index values were significantly higher than the other clinical variables. Finally, ROC curve analysis revealed that the AUC value of 0.731 for the NerRLsig was greater than as compared to traditional clinical indicators (Figure 5(g)). Altogether, the above results demonstrated that the NerRLsig could be employed robustly and accurately to predict clinical outcomes in BC patients. Additionally, the bar and scatter plots revealed that the T status (*p* < 0.05) and clinical stage (*p* < 0.01) were notably associated with the NerRLsig (Figure 6(a) and Supplementary Table 6). Figures 6(b)–6(h) show the differences in risk scores between subgroups with varied clinical characteristics, with statistically significant differences in risk scores within the clinical stage (*p* = 0.017), T status (*p* = 0.025), and N status (*p* = 0.028).

### 3.4. Functional Signaling Exploration of the NerRLsig

**Figure 7:**
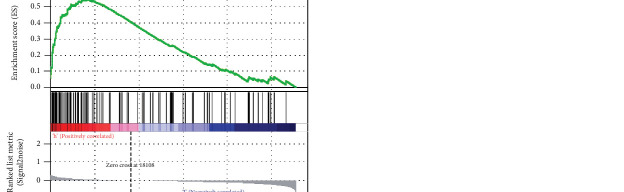
Functional analysis of the necroptosis-related lncRNA signature (NerRLsig). Representative results of GO (a) and KEGG analyses (b) of the NerRLsig. (c–h) Gene set enrichment analysis (GSEA) of the high-risk group based on the NerRLsig.

To study the underlying action mechanisms of the NerRLsig in BC, DEGs between the two subgroups were screened and GO analysis indicated that the DEGs were primarily focused on glycosaminoglycan binding, collagen-containing extracellular matrix, and epidermis development (Figure 7(a)). KEGG analysis revealed that the DEGs are mainly engaged in neuroactive ligand-receptor interaction, PI3K-Akt signaling pathway, calcium signaling pathway, focal adhesion, and proteoglycans in cancer (Figure 7(b)). Additionally, GSEA results demonstrated that focal adhesion, pathways in cancer, WNT signaling pathway, TGF_BETA signaling pathway, ECM receptor interaction, and leukocyte transendothelial migration were remarkably enriched in the high-NerRLsig group (Figures 7(c)–7(h) and Supplementary Table 7).

### 3.5. Immunity and Mutation Analyses of the NerRLsig

**Figure 8:**
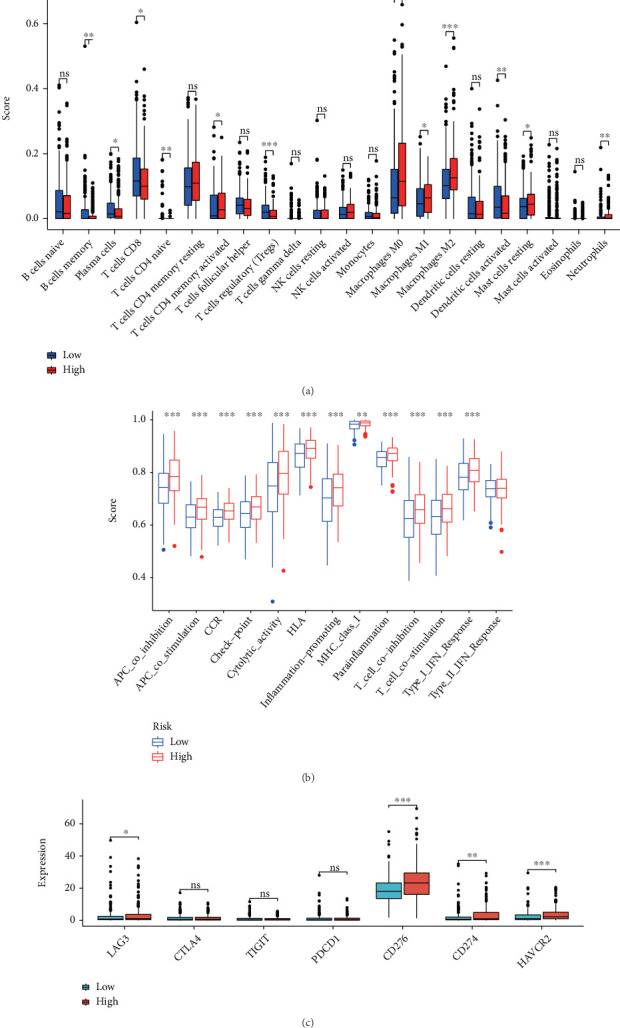
Immune landscape of the necroptosis-related lncRNA signature (NerRLsig). (a) The different proportions of tumor-infiltrating cells between the high-risk group and the low-risk group. (b) 13 immune-related pathways in the high-risk group and the low-risk group are displayed in boxplots. (c) The expression levels of immune checkpoint molecules in the high-risk group and the low-risk group.

**Figure 9:**
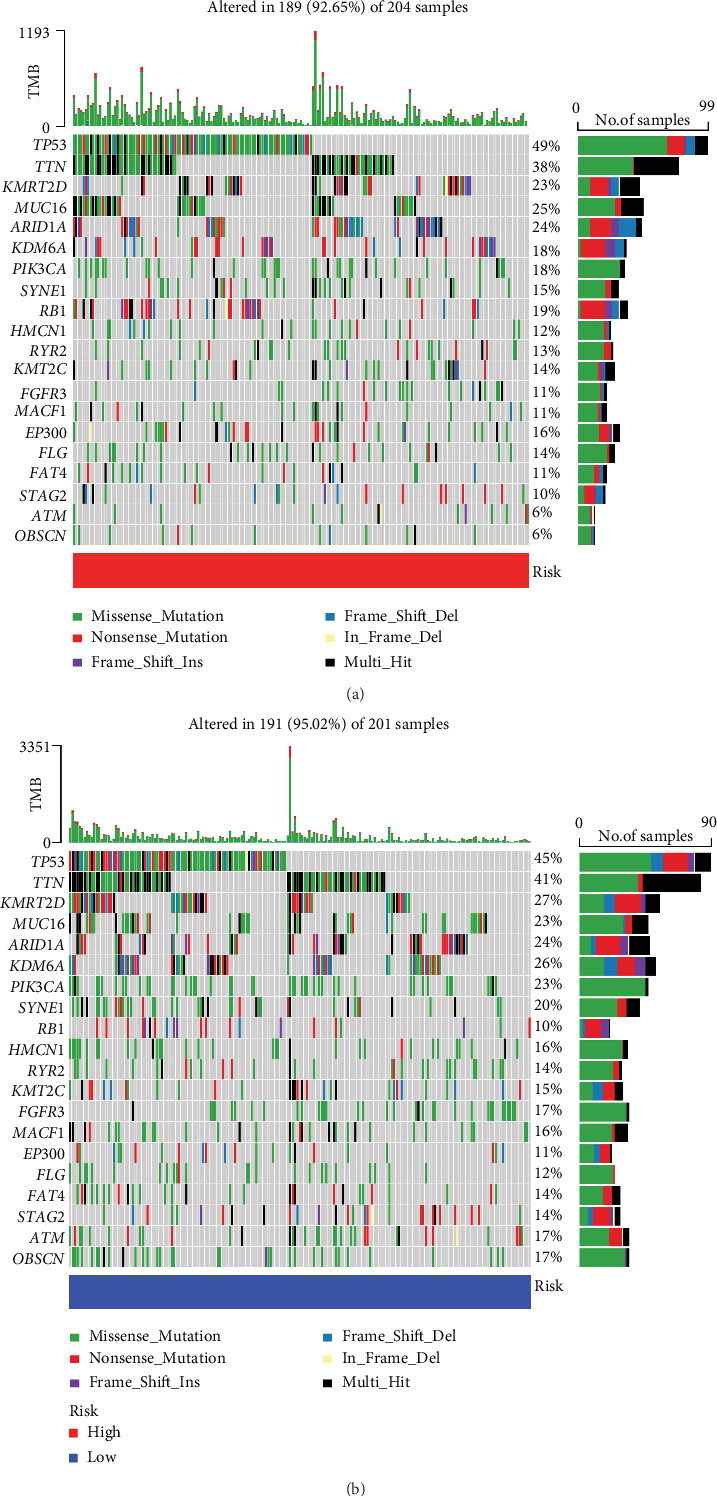
Somatic mutation of the necroptosis-related lncRNA signature (NerRLsig). (a, b) Oncoplots of the mutated genes in the (a) high-NerRLsig and (b) low-NerRLsig subgroups of the TCGA cohort.

To ascertain whether the NerRLsig was related to tumor immunity, differences in immune infiltrating cells between the low-NerRLsig and high-NerRLsig subgroups were compared and the results are illustrated in Figure 8(a). Activated memory CD4 T cells, M0 macrophages, M1 macrophages, M2 macrophages, and resting mast cells were demonstrated to be upregulated in the high-NerRLsig subgroup. In contrast, B cell memory, plasma cells, CD8 T cells, naïve CD4 T cells, regulatory T cells (Tregs), activated dendritic cells, and neutrophils were shown to be upregulated in the low-NerRLsig group. The ssGSEA analysis identified differences between the low-NerRLsig and the high-NerRLsig groups in terms of several immune-related pathways, such as checkpoint, cytolytic activity, and type II IFN response (Figure 8(b)). In addition, we compared the differences in the expression levels of immune checkpoints between two subgroups. Results showed that patients in the high-NerRLsig group have elevated LAG3 expression (*p* < 0.05), HAVCR2 (*p* < 0.001), CD274 (*p* < 0.01), CD276 (*p* < 0.001), PDCD1 (*p* > 0.05), TIGIT (*p* > 0.05), and CTLA4 (*p* > 0.05), while the latter three were not statistically different (Figure 8(c)). Taken together, the above results suggested that the immune status was different between the low-NerRLsig and high-NerRLsig subgroups, which could provide insight into the development of tumor immunotherapy for BC. Additionally, the waterfall plot showed mutational landscape in the high-NerRLsig and low-NerRLsig subgroups (Figures 9(a) and 9(b)). Genes such as TP53 (49%), TTN (38%), MUC16 (25%), ARID1A (24%), and KMT2D (23%) showed five major mutation frequencies in the high-NerRLsig subgroup, while TP53 (45%), TTN (41%), KMT2D (27%), KDM6A (26%), and ARID1A (24%) were the five most frequently mutated genes in the low-NerRLsig subgroup.

### 3.6. Chemotherapy Efficacy Related to the NerRLsig

**Figure 10:**
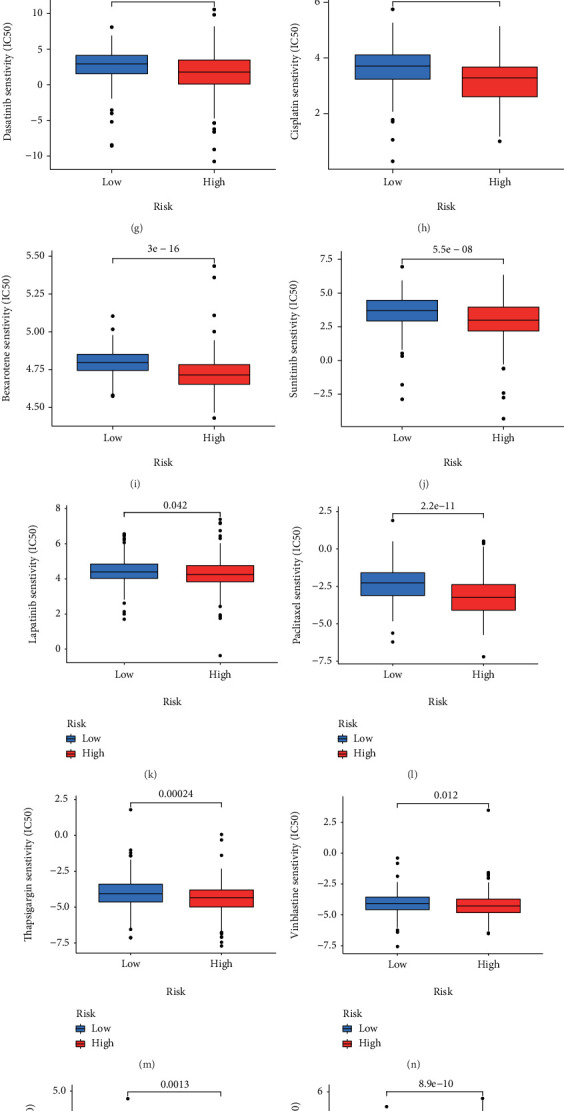
Analysis of chemotherapeutic susceptibility. (a–p) Sensitivity to different types of chemotherapeutic agents. (q) The relationship between MAP3K14-AS1 and STAG3L5P-PVRIG2P-PILRB with drug sensitivity.

In addition to immune checkpoint blockades therapy, we also attempted to explore whether there was an association between the NerRLsig and sensitivity of BC patients to conventional chemotherapeutic agents and common targeted drugs. Results revealed that patients in the low-NerRLsig subgroup patients had lower IC50 values for methotrexate (Figure 10(a), *p* < 0.001) and axitinib (Figure 10(b), *p* < 0.01). In contrast, a high risk score was associated with a lower IC50 values of chemotherapeutics such as bicalutamide (Figure 10(c), *p* < 0.001), bleomycin (Figure 10(d), *p* < 0.001), imatinib (Figure 10(e), *p* < 0.001), docetaxel (Figure 10(f), *p* < 0.001), dasatinib (Figure 10(g), *p* < 0.001), cisplatin (Figure 10(h), *p* < 0.001), bexarotene (Figure 10(i), *p* < 0.001), sunitinib (Figure 10(j), *p* < 0.001), lapatinib (Figure 10(k), *p* < 0.05), paclitaxel (Figure 10(l), *p* < 0.001), thapsigargin (Figure 10(m), *p* < 0.001), vinblastine (Figure 10(n), *p* < 0.05), sorafenib (Figure 10(o), *p* < 0.01), and pazopanib (Figure 10(p), *p* < 0.001). Additionally, we obtained the 17 drugs with the most statistically significant differences by performing separate drug sensitivity analyses for lncRNA in the prognostic model (Figure 10(q) and Supplementary Table 8). The results demonstrated that MAP3K14-AS1 expression had a positive correlation with the sensitivity of erlotinib, afatinib, neratinib, gefitinib, temsirolimus, dacomitinib, everolimus, lapatinib, and ibrutinib, while was negatively correlated the sensitivity of selumetinib, ARRY-162, encorafenib, pipamperone, cobimetinib (isomer 1), vemurafenib, and carmustine. In addition, the higher the expression of STAG3L5P-PVRIG2P-PILRB in BC patients, the higher the sensitivity of patients to decitabine.

## 4. Discussion

BC is a highly malignant cancer with a poor prognosis and remains difficult to treat [2]. Although multimodality treatments have allowed patients to live longer, treatment outcomes remain poor [25]. In addition, patients with similar clinical features may also present with different prognoses and responses to oncological treatment [34]. Effective and individualized treatments must be researched for BC patients. Herein, we created a prognostic NerRLsig to predict OS in BC patients. High-NerRLsig was associated with worse clinical outcomes and certain oncogenic signaling pathways. Additionally, by combining risk scores with other clinical features, we created a nomogram that has the power to predict OS in patients with BC in the TCGA dataset. Finally, we found differences in immune composition, mutational burden, and chemotherapeutic drug sensitivity between the low-NerRLsig and high-NerRLsig subgroups.

In previous studies, prognostic lncRNA models have been established in BC. For instance, a nine-ferroptosis-related lncRNA signature and a four-pyroptosis-related lncRNA signature have been developed to assess the risk of BLCA patients and to inform clinical treatment [35, 36]. It is therefore important to identify a prognostic NerRLsig in BC. Here, 12 DEnrlncRNAs (AC015802.4, AL391807.1, AL078644.1, AC023825.2, AL132655.2, AP003352.1, STAG3L5P-PVRIG2P-PILRB, AC024451.4, MAP3K14-AS1, AL731567.1, AC010542.5, and AC009299.2) were identified to construct a predictive signature. A recent report indicated that significantly higher levels of MAP3K14-AS1 methylation were observed in tumor tissues compared to normal colorectal cancer tissues [37]. However, the biological role of most of them in tumorigenesis remains to be elucidated.

The crucial roles of immune cells in the tumor microenvironment have been confirmed. For example, CD8+ T cells were considered to be the main driver of anti-tumor immunity, and CD8+ T cell exhaustion usually led to ineffective control of persistent infections and cancers [38]. A recent report demonstrated that dysregulation of the immune system and inflammatory factor storm were common features of high-risk NMIBC and COVID-19 [39]. M2 macrophages have been demonstrated to promote tumor invasion and metastasis by upregulating anti-inflammatory cytokines and chemokines or to hinder the efficacy of chemotherapy and radiotherapy by suppressing CD8+ T cell function, leading to tumor progression and poor outcomes [40]. In this work, we found lower CD8 T cells and higher M2 macrophage infiltration in the high-NerRLsig group, suggesting fewer numbers of infiltrated antitumor immune effector cells and the stronger infiltrated inhibitory immune cells may result in poor overcomes of patients in the high-NerRLsig subgroup. Paradoxically, we also observed lower regulatory T cells (Tregs) and increased M1 macrophages infiltration in the high-NerRLsig subgroup, with the former playing a negative role in triggering an effective antitumor immune response and the latter playing a positive role [41, 42]. The above results indicated that the regulatory role of necroptosis-associated lncRNA in tumor immunity may be bidirectional and complex; further analyses were necessary to confirm their roles in immune regulation. Immune checkpoint inhibitors exert immunosuppressive effects by inhibiting the generation of protective immunity [43]. The results revealed upregulated expression of immune checkpoints in the high-NerRLsig subgroup, which further suggested that ICI treatment may be more effective in the high-NerRLsig subgroup.

Moreover, we studied the relationship between the signature and chemotherapy drug response to facilitate personalized treatment decisions. We noted that patients in the low-NerRLsig group were more sensitive to methotrexate and axitinib, while patients in the high-NerRLsig group were more sensitive to bicalutamide, bleomycin, imatinib, docetaxel, dasatinib, cisplatin, bexarotene, sunitinib, lapatinib, paclitaxel, thapsigargin, vinblastine, sorafenib, and pazopanib. Additionally, increased MAP3K14-AS1 expression was associated with cancer cell resistance to erlotinib, afatinib, neratinib, gefitinib, temsirolimus, dacomitinib, everolimus, lapatinib, and ibrutinib, and increased expression of STAG3L5P-PVRIG2P-PILRB was associated with cancer cell sensitivity to decitabine. These data suggested that MAP3K14-AS1 and STAG3L5P-PVRIG2P-PILRB could be used exploited as therapeutic targets for drug design or adjuvant therapy.

Indubitably, the potential limitations of our study cannot be ignored. Firstly, the NerRLsig was built and validated based on a publicly available database, more prospective clinical data is needed to determine its clinical implications. Secondly, experimental verification on hub nrlncRNAs is needed. Finally, the response to immunotherapy and chemotherapy needs to be further validated with clinical data from other cohorts.

In conclusion, the research emphasized the prognostic significance of the NerRLsig in BC. Clinical outcomes, genetic variants, functional pathways, immunological heterogeneity, and drug responses of the prognostic signature were also uncovered.

## Data Availability

The public database mentioned in this study is publicly available for reanalyzing.

## Supplementary Materials

Supplementary 1 Supplementary Table 1: necroptosis-related genes (NRGs) and lncRNAs.

Supplementary 2 Supplementary Table 2: differentially expressed necroptosis-related lncRNAs.

Supplementary 3 Supplementary Table 3: 42 differentially expressed necroptosis-related lncRNAs were identified by univariate Cox analysis.

Supplementary 4 Supplementary Table 4: 12 prognosis-related differentially expressed necroptosis-related lncRNAs.

Supplementary 5 Supplementary Table 5: univariate and multivariate Cox regression analyses for the NerRLsig as an independent prognostic factor.

Supplementary 6 Supplementary Table 6: the risk score correlated with clinicopathological features in bladder cancer.

Supplementary 7 Supplementary Table 7: gene set enrichment analysis (GSEA) of high-risk group based on the necroptosis-related lncRNA signature (NerRLsig).

Supplementary 8 Supplementary Table 8: the relationship between MAP3K14-AS1 and STAG3L5P-PVRIG2P-PILRB with drug sensitivity.

## References

[1] Kirkali Z., Chan T., Manoharan M. (2005). Bladder cancer: epidemiology, staging and grading, and diagnosis. *Urology*.

[2] Grayson M. (2017). Bladder cancer. *Nature*.

[3] Dobruch J., Daneshmand S., Fisch M. (2016). Gender and bladder cancer: a collaborative review of etiology, biology, and outcomes. *European Urology*.

[4] Morgan T. M., Keegan K. A., Clark P. E. (2011). Bladder cancer. *Current Opinion in Oncology*.

[5] Patel V. G., Oh W. K., Galsky M. D. (2020). Treatment of muscle-invasive and advanced bladder cancer in 2020. *CA: a Cancer Journal for Clinicians*.

[6] Lenis A. T., Lec P. M., Chamie K., Mshs M. D. (2020). Bladder Cancer. *Jama*.

[7] Gong Y., Fan Z., Luo G. (2019). The role of necroptosis in cancer biology and therapy. *Molecular cancer*.

[8] Bertheloot D., Latz E., Franklin B. S. (2021). Necroptosis, pyroptosis and apoptosis: an intricate game of cell death. *Cellular & molecular immunology*.

[9] Frank D., Vince J. E. (2019). Pyroptosis versus necroptosis: similarities, differences, and crosstalk. *Cell Death and Differentiation*.

[10] Khoury M. K., Gupta K., Franco S. R., Liu B. (2020). Necroptosis in the pathophysiology of disease. *The American Journal of Pathology*.

[11] Han Q., Ma Y., Wang H. (2018). Resibufogenin suppresses colorectal cancer growth and metastasis through RIP3-mediated necroptosis. *Journal of translational medicine*.

[12] Schneider A. T., Gautheron J., Feoktistova M. (2017). RIPK1 suppresses a TRAF2-dependent pathway to liver cancer. *Cancer Cell*.

[13] Yang H., Ma Y., Chen G. (2016). Contribution of RIP3 and MLKL to immunogenic cell death signaling in cancer chemotherapy. *Oncoimmunology*.

[14] Wang Y., Hao F., Nan Y. (2018). PKM2 inhibitor shikonin overcomes the cisplatin resistance in bladder cancer by inducing necroptosis. *International Journal of Biological Sciences*.

[15] Jarroux J., Morillon A., Pinskaya M. (2017). History, discovery, and classification of lncRNAs. *Advances in Experimental Medicine and Biology*.

[16] Elguindy M. M., Mendell J. T. (2021). _NORAD_ -induced Pumilio phase separation is required for genome stability. *Nature*.

[17] Sun Q., Hao Q., Prasanth K. V. (2018). Nuclear long noncoding RNAs: key regulators of gene expression. *Trends in Genetics*.

[18] Schmitz S. U., Grote P., Herrmann B. G. (2016). Mechanisms of long noncoding RNA function in development and disease. *Cellular and Molecular Life Sciences*.

[19] Logotheti S., Marquardt S., Gupta S. K. (2020). LncRNA-SLC16A1-AS1 induces metabolic reprogramming during bladder cancer progression AS target and co-activator of E2F1. *Theranostics*.

[20] Chen C., He W., Huang J. (2018). _LNMAT1_ promotes lymphatic metastasis of bladder cancer via CCL2 dependent macrophage recruitment. *Nature communications*.

[21] Tran D. D. H., Kessler C., Niehus S. E., Mahnkopf M., Koch A., Tamura T. (2018). Myc target gene, long intergenic noncoding RNA, _Linc00176_ in hepatocellular carcinoma regulates cell cycle and cell survival by titrating tumor suppressor microRNAs. *Oncogene*.

[22] Huang Y., Feng Y., Cui L. (2021). Autophagy-related LC3 accumulation interacted directly with LIR containing RIPK1 and RIPK3, stimulating necroptosis in hypoxic cardiomyocytes. *Frontiers in Cell and Development Biology*.

[23] Zhang M., Wang N., Song P. (2020). LncRNA GATA3-AS1 facilitates tumour progression and immune escape in triple- negative breast cancer through destabilization of GATA3 but stabilization of PD-L1. *Cell proliferation*.

[24] Chen Y. G., Satpathy A. T., Chang H. Y. (2017). Gene regulation in the immune system by long noncoding RNAs. *Nature immunology*.

[25] Dobruch J., Oszczudłowski M. (2021). Bladder Cancer: Current Challenges and Future Directions. *Medicina (Kaunas)*.

[26] Daneshmand S. (2020). Bladder cancer: advances and innovations. *European Urology Focus*.

[27] Ma G., Yang X., Liang Y. (2018). Precision medicine and bladder cancer heterogeneity. *Bulletin du Cancer*.

[28] Li Z., Li Y., Zhong W., Huang P. (2021). m6A-related lncRNA to develop prognostic signature and predict the immune landscape in bladder cancer. *Journal of oncology*.

[29] Cao R., Yuan L., Ma B., Wang G., Tian Y. (2021). Tumour microenvironment (TME) characterization identified prognosis and immunotherapy response in muscle-invasive bladder cancer (MIBC). *Cancer Immunology, Immunotherapy*.

[30] Yang L., Li C., Qin Y. (2021). A novel prognostic model based on ferroptosis-related gene signature for bladder cancer. *Frontiers in Oncology*.

[31] Tomczak K., Czerwinska P., Wiznerowicz M. (2015). The Cancer Genome Atlas (TCGA): an immeasurable source of knowledge. *Contemporary oncology*.

[32] Yang W., Soares J., Greninger P. (2013). Genomics of Drug Sensitivity in Cancer (GDSC): a resource for therapeutic biomarker discovery in cancer cells. *Nucleic Acids Research*.

[33] Shankavaram U. T., Varma S., Kane D. (2009). CellMiner: a relational database and query tool for the NCI-60 cancer cell lines. *BMC Genomics*.

[34] Tran L., Xiao J. F., Agarwal N., Duex J. E., Theodorescu D. (2021). Advances in bladder cancer biology and therapy. *Nature Reviews Cancer*.

[35] Chen M., Nie Z., Li Y. (2021). A new Ferroptosis-related lncRNA signature predicts the prognosis of bladder cancer patients. *Frontiers in Cell and Development Biology*.

[36] Lu Z., Tang F., Li Z. (2022). Prognosis risk model based on pyroptosis-related lncRNAs for bladder cancer. *Disease Markers*.

[37] Barault L., Amatu A., Siravegna G. (2018). Discovery of methylated circulating DNA biomarkers for comprehensive non-invasive monitoring of treatment response in metastatic colorectal cancer. *Gut*.

[38] Farhood B., Najafi M., Mortezaee K. (2019). CD8+ cytotoxic T lymphocytes in cancer immunotherapy: a review. *Journal of cellular physiology*.

[39] Busetto G. M., Porreca A., del Giudice F. (2020). SARS-CoV-2 infection and high-risk non-muscle-invasive bladder cancer: are there any common features?. *Urologia Internationalis*.

[40] Valeta-Magara A., Gadi A., Volta V. (2019). Inflammatory breast cancer promotes development of M2 tumor-associated macrophages and cancer mesenchymal cells through a complex chemokine network. *Cancer research*.

[41] Takeuchi Y., Nishikawa H. (2016). Roles of regulatory T cells in cancer immunity. *International Immunology*.

[42] Mantovani A., Marchesi F., Malesci A., Laghi L., Allavena P. (2017). Tumour-associated macrophages as treatment targets in oncology. *Nature Reviews. Clinical Oncology*.

[43] Li B., Chan H. L., Chen P. (2019). Immune checkpoint inhibitors: basics and challenges. *Current Medicinal Chemistry*.

