# Supplementary material for: Comprehensive Characterization of Necroptosis-Related lncRNAs in Bladder Cancer Identifies a Novel Signature for Prognosis Prediction
Source: Dis Markers. 2022 Jun 6;2022:2360299. doi: 10.1155/2022/2360299 (PMC9194958; doi:10.1155/2022/2360299)
Supplement: Supplementary 1 — Supplementary Table 1: necroptosis-related genes (NRGs) and lncRNAs. [file 2360299.f1.pdf]

Gene	Type
AC092301.1	lncRNA
AL133338.1	lncRNA
AC002128.1	lncRNA
AL109955.1	lncRNA
AC104825.1	lncRNA
AC006042.1	lncRNA
AC092611.1	lncRNA
MCF2L-AS1	lncRNA
AC025766.1	lncRNA
AL121820.1	lncRNA
AC087286.2	lncRNA
AC073957.3	lncRNA
AC084018.2	lncRNA
CDKN2A-DT	lncRNA
AL353622.2	lncRNA
AL133467.1	lncRNA
AC024267.3	lncRNA
AL161891.1	lncRNA
AC124312.2	lncRNA
AP000766.1	lncRNA
AC104785.1	lncRNA
AC074117.1	lncRNA
AC040162.3	lncRNA
DNM3OS	lncRNA
AL355574.1	lncRNA
AC025031.1	lncRNA
CH17-340M24.3	lncRNA
AC008764.6	lncRNA
AC023908.3	lncRNA
AC018809.1	lncRNA
AC027449.1	lncRNA
AL159169.3	lncRNA
AL021707.4	lncRNA
AL158166.1	lncRNA
AC005790.1	lncRNA
AC104532.2	lncRNA
MIR1-1HG-AS1	lncRNA
AC092803.1	lncRNA
AC104170.1	lncRNA
AL357054.4	lncRNA
AC009095.1	lncRNA

AL096870.2	lncRNA
AC025280.1	lncRNA
AL136038.4	lncRNA
TFAP2A-AS1	lncRNA
RMRP	lncRNA
NCBP2-AS1	lncRNA
AC015802.4	lncRNA
AC018766.1	lncRNA
AC027307.3	lncRNA
AL117335.1	lncRNA
LINC01322	lncRNA
YEATS2-AS1	lncRNA
MHENCR	lncRNA
AC079160.1	lncRNA
AC026740.1	lncRNA
AC087521.1	lncRNA
AC108463.2	lncRNA
AL391807.1	lncRNA
LINC00641	lncRNA
AL133355.1	lncRNA
LINC02195	lncRNA
LINC00426	lncRNA
CASC2	lncRNA
AC018926.2	lncRNA
LINC02489	lncRNA
ODF2-AS1	lncRNA
AL023284.4	lncRNA
AL139397.1	lncRNA
MIR497HG	lncRNA
AC107308.1	lncRNA
AC009754.1	lncRNA
AC007128.2	lncRNA
AC092127.2	lncRNA
AC124068.2	lncRNA
LINC01124	lncRNA
RAB11B-AS1	lncRNA
AL662844.4	lncRNA
AL133346.1	lncRNA
AC018653.3	lncRNA
AC107959.1	lncRNA
LINC00910	lncRNA
AP001793.1	lncRNA
AC036108.3	lncRNA
MCCC1-AS1	lncRNA

LINC01550	lncRNA
AC007622.2	lncRNA
AP006621.3	lncRNA
AP001107.1	lncRNA
AC008608.2	lncRNA
AL365361.1	lncRNA
AL441992.1	lncRNA
TMPO-AS1	lncRNA
AL390067.1	lncRNA
LINC02384	lncRNA
AP005329.1	lncRNA
MIR4435-2HG	lncRNA
AL670729.1	lncRNA
AC024075.3	lncRNA
AC092376.2	lncRNA
GATA3-AS1	lncRNA
AC005387.1	lncRNA
AP003071.4	lncRNA
AP003086.2	lncRNA
AC139887.1	lncRNA
THAP9-AS1	lncRNA
KDM4A-AS1	lncRNA
WASHC5-AS1	lncRNA
LINC01781	lncRNA
RASSF8-AS1	lncRNA
MAGEA10-MAGEA5	lncRNA
MIR600HG	lncRNA
AC069281.2	lncRNA
LINC00885	lncRNA
XXYLT1-AS2	lncRNA
AL078644.1	lncRNA
AC108449.2	lncRNA
AL132780.1	lncRNA
ARRDC1-AS1	lncRNA
AL133415.1	lncRNA
U62317.1	lncRNA
AC068790.7	lncRNA
AC053503.4	lncRNA
AL691482.3	lncRNA
LINC00894	lncRNA
SNHG7	lncRNA
KRT7-AS	lncRNA
AP003392.4	lncRNA
AL031709.1	lncRNA

AC129510.1	lncRNA
AL118506.1	lncRNA
AC024075.1	lncRNA
MEG9	lncRNA
AC138932.1	lncRNA
LINC00941	lncRNA
AL139041.1	lncRNA
AP001625.2	lncRNA
AC079921.2	lncRNA
AC009093.6	lncRNA
CRTC3-AS1	lncRNA
C8orf44	lncRNA
FAM218A	lncRNA
AC009065.4	lncRNA
SH3RF3-AS1	lncRNA
AC090825.1	lncRNA
AC005104.1	lncRNA
LINC01081	lncRNA
AL161452.1	lncRNA
AC008764.8	lncRNA
TRAF3IP2-AS1	lncRNA
ADAMTS9-AS1	lncRNA
AC008735.2	lncRNA
AC079313.2	lncRNA
AC007292.1	lncRNA
AC008537.2	lncRNA
AC010618.2	lncRNA
ANKRD10-IT1	lncRNA
LINC01405	lncRNA
MAGI2-AS3	lncRNA
LINC01119	lncRNA
AF117829.1	lncRNA
AL121652.1	lncRNA
AC013553.3	lncRNA
AC244034.2	lncRNA
AC016888.1	lncRNA
OSMR-AS1	lncRNA
AC023825.2	lncRNA
ZKSCAN2-DT	lncRNA
AC009041.2	lncRNA
ZNF252P-AS1	lncRNA
AC005785.1	lncRNA
AF001548.1	lncRNA
AC005332.1	lncRNA

AC010998.2	lncRNA
DHRS4-AS1	lncRNA
AL121929.2	lncRNA
MYOSLID	lncRNA
NRIR	lncRNA
DUBR	lncRNA
AC015849.4	lncRNA
CEBPA-DT	lncRNA
AC019131.2	lncRNA
AL132655.2	lncRNA
AC126755.1	lncRNA
ZNF32-AS2	lncRNA
AC007128.1	lncRNA
HM13-IT1	lncRNA
AC002398.2	lncRNA
AC116407.2	lncRNA
LINC01213	lncRNA
LINC01675	lncRNA
AL157871.5	lncRNA
AC020978.7	lncRNA
AC027601.3	lncRNA
LINC01139	lncRNA
AC018529.1	lncRNA
AC073046.1	lncRNA
AP003352.1	lncRNA
AC006435.2	lncRNA
AC021087.1	lncRNA
AL731577.2	lncRNA
MAL2-AS1	lncRNA
AC073869.1	lncRNA
AL163953.1	lncRNA
AC099850.3	lncRNA
AL118511.1	lncRNA
AC144548.1	lncRNA
AC002398.1	lncRNA
AC010326.3	lncRNA
AP4B1-AS1	lncRNA
AL390728.4	lncRNA
AC136475.2	lncRNA
AC092910.3	lncRNA
LINC02285	lncRNA
AC011933.3	lncRNA
LINC02109	lncRNA
SPAG5-AS1	lncRNA

LINC02361	lncRNA
AC010976.2	lncRNA
LINC01411	lncRNA
AC135507.1	lncRNA
AC012073.1	lncRNA
AC005332.7	lncRNA
AP000892.3	lncRNA
LINC02084	lncRNA
LINC01126	lncRNA
LINC01353	lncRNA
AC092811.1	lncRNA
AL390294.1	lncRNA
AC024361.3	lncRNA
ACTA2-AS1	lncRNA
LINC01355	lncRNA
AL133371.2	lncRNA
LINC02577	lncRNA
STAG3L5P-PVRIG2P-PILRB	lncRNA
AC005070.3	lncRNA
CCDC183-AS1	lncRNA
AC090589.3	lncRNA
FGF12-AS3	lncRNA
AC139887.2	lncRNA
AC004449.1	lncRNA
PSPC1-AS2	lncRNA
AC018752.1	lncRNA
AC017002.3	lncRNA
AC004943.2	lncRNA
LINC02352	lncRNA
FTX	lncRNA
AL592424.1	lncRNA
AL512770.1	lncRNA
SNHG10	lncRNA
CDKN2B-AS1	lncRNA
AC017104.1	lncRNA
AL450998.2	lncRNA
CHL1-AS2	lncRNA
AC024451.4	lncRNA
NDUFB2-AS1	lncRNA
A2M-AS1	lncRNA
AL928654.2	lncRNA
HAND2-AS1	lncRNA
TRG-AS1	lncRNA

AL161772.1	lncRNA
AL590729.1	lncRNA
AC010168.2	lncRNA
LINC01820	lncRNA
AC008735.1	lncRNA
LINC01004	lncRNA
GHRLOS	lncRNA
AC005180.1	lncRNA
AL117329.1	lncRNA
AC026401.3	lncRNA
AC091057.1	lncRNA
NBR2	lncRNA
LINC02100	lncRNA
FLJ12825	lncRNA
AC011477.3	lncRNA
AC027601.1	lncRNA
ZFHX4-AS1	lncRNA
AC245060.6	lncRNA
AC007637.1	lncRNA
AC008735.4	lncRNA
AC011461.1	lncRNA
MORF4L2-AS1	lncRNA
AL513165.1	lncRNA
AC010491.1	lncRNA
TGFB2-AS1	lncRNA
AC015922.2	lncRNA
AC008750.1	lncRNA
AL139352.1	lncRNA
AC027097.2	lncRNA
AC010247.2	lncRNA
ATP2A1-AS1	lncRNA
AC022893.1	lncRNA
LINC02241	lncRNA
HHIP-AS1	lncRNA
AC006557.1	lncRNA
AL032819.1	lncRNA
AC106897.1	lncRNA
C1orf220	lncRNA
GNG12-AS1	lncRNA
AC099482.1	lncRNA
AC092802.2	lncRNA
AC125257.1	lncRNA
AC006369.1	lncRNA
SNRK-AS1	lncRNA

AC027682.4	lncRNA
NIFK-AS1	lncRNA
LENG8-AS1	lncRNA
AL604028.1	lncRNA
AC134043.2	lncRNA
LINC01833	lncRNA
CARMN	lncRNA
RUSC1-AS1	lncRNA
MEG3	lncRNA
AC010503.4	lncRNA
LINC00861	lncRNA
MANEA-DT	lncRNA
AC025031.4	lncRNA
MBNL1-AS1	lncRNA
AC106864.1	lncRNA
LINC01936	lncRNA
AC092119.2	lncRNA
CACNA1C-AS2	lncRNA
AC008555.1	lncRNA
ZNF436-AS1	lncRNA
PCAT7	lncRNA
AC105339.2	lncRNA
MAP3K14-AS1	lncRNA
NKILA	lncRNA
AP006621.2	lncRNA
AC127024.4	lncRNA
AC005330.1	lncRNA
AL590652.1	lncRNA
LINC01342	lncRNA
AC034102.8	lncRNA
AL731567.1	lncRNA
AP001462.1	lncRNA
AC093788.1	lncRNA
PCAT19	lncRNA
AC080129.2	lncRNA
NPTN-IT1	lncRNA
GAS6-DT	lncRNA
AC024361.1	lncRNA
LINC01719	lncRNA
U62317.2	lncRNA
AC092123.1	lncRNA
WASIR2	lncRNA
AC010976.1	lncRNA
AC010201.2	lncRNA

AL449423.1	lncRNA
AC020928.1	lncRNA
AL161729.4	lncRNA
STAM-AS1	lncRNA
AC124944.3	lncRNA
AL157394.1	lncRNA
AC109460.2	lncRNA
AC012615.6	lncRNA
SOX1-OT	lncRNA
AP001189.1	lncRNA
AC090152.1	lncRNA
FOXD3-AS1	lncRNA
EMX2OS	lncRNA
LINC02178	lncRNA
C9orf163	lncRNA
AC106881.1	lncRNA
AC010542.5	lncRNA
AL450384.1	lncRNA
LINC01140	lncRNA
AC108471.2	lncRNA
AC067852.3	lncRNA
TTLL11-IT1	lncRNA
AP000866.5	lncRNA
AC119396.1	lncRNA
AC008543.1	lncRNA
HDAC2-AS2	lncRNA
AP002761.1	lncRNA
AC005519.1	lncRNA
LINC01775	lncRNA
AP003071.3	lncRNA
AC092809.4	lncRNA
PAXIP1-AS2	lncRNA
PPP1R26-AS1	lncRNA
PP7080	lncRNA
LINC01134	lncRNA
BX322562.1	lncRNA
AC138956.2	lncRNA
LINC00173	lncRNA
FENDRR	lncRNA
LINC01852	lncRNA
VIM-AS1	lncRNA
CYTOR	lncRNA
AC006042.3	lncRNA
MIR100HG	lncRNA

AC021242.3	lncRNA
GAS8-AS1	lncRNA
AL021707.8	lncRNA
NARF-IT1	lncRNA
AC020663.2	lncRNA
AL031282.2	lncRNA
AC245052.4	lncRNA
AL354993.2	lncRNA
LINC01914	lncRNA
AC025287.3	lncRNA
DANCR	lncRNA
AL445423.1	lncRNA
AC092171.5	lncRNA
AC104971.1	lncRNA
AC009148.1	lncRNA
DCST1-AS1	lncRNA
MAP3K20-AS1	lncRNA
AL117350.1	lncRNA
AL158166.2	lncRNA
FMR1-IT1	lncRNA
AL596244.1	lncRNA
AP000866.1	lncRNA
AC024075.2	lncRNA
AC093278.2	lncRNA
AL132642.1	lncRNA
AP001619.1	lncRNA
AL583785.1	lncRNA
LINC02156	lncRNA
U62631.1	lncRNA
AL121832.3	lncRNA
LINC00456	lncRNA
AC010761.4	lncRNA
LINC02202	lncRNA
AC012645.2	lncRNA
LINC01943	lncRNA
AC234775.3	lncRNA
LINC01410	lncRNA
LINC01082	lncRNA
CAPN10-DT	lncRNA
C1RL-AS1	lncRNA
LINC00630	lncRNA
AP001107.5	lncRNA
AC244093.5	lncRNA
CEP83-DT	lncRNA

AC087294.1	lncRNA
AC004253.1	lncRNA
LINC01215	lncRNA
AL354892.2	lncRNA
AL158212.3	lncRNA
LINC00702	lncRNA
AC008764.2	lncRNA
AL136295.2	lncRNA
AP002336.2	lncRNA
LINC01352	lncRNA
CDC42-IT1	lncRNA
AC022150.2	lncRNA
AC016876.3	lncRNA
AC020911.1	lncRNA
AP001189.3	lncRNA
AL157838.1	lncRNA
SSBP3-AS1	lncRNA
SNHG9	lncRNA
AL135999.1	lncRNA
AC002310.1	lncRNA
AC015819.1	lncRNA
AL365330.1	lncRNA
AC004034.1	lncRNA
AC005180.2	lncRNA
LINC00900	lncRNA
AC073052.1	lncRNA
PTPRG-AS1	lncRNA
LINC02584	lncRNA
LINC02588	lncRNA
AC008543.3	lncRNA
LINC02104	lncRNA
AC021491.2	lncRNA
FBXL19-AS1	lncRNA
AC090559.1	lncRNA
SNHG26	lncRNA
LINC00892	lncRNA
AC092794.1	lncRNA
AL592211.1	lncRNA
AL139393.2	lncRNA
AC083906.3	lncRNA
AC015849.3	lncRNA
MCM3AP-AS1	lncRNA
AC084125.2	lncRNA
AC021683.1	lncRNA

Z94721.1	lncRNA
AC009299.2	lncRNA
AC010271.2	lncRNA
AC007384.1	lncRNA
AL138756.1	lncRNA
AL356019.2	lncRNA
TSPOAP1-AS1	lncRNA
NARF-AS1	lncRNA
AC124312.3	lncRNA
AL442128.2	lncRNA
AC078795.1	lncRNA
AC108860.2	lncRNA
AC100778.2	lncRNA
AL138995.1	lncRNA
AC013403.2	lncRNA
AC127070.1	lncRNA
AL109741.1	lncRNA
AF131215.5	lncRNA
AL445490.1	lncRNA
ZNF32-AS1	lncRNA
AC005253.1	lncRNA
ACTN1-AS1	lncRNA
AP001628.1	lncRNA
AC132192.2	lncRNA
TSC1	NRG
MAP3K7	NRG
BRAF	NRG
BCL2	NRG
FLT3	NRG
BACH2	NRG
GATA3	NRG
IDH1	NRG
RNF31	NRG
KLF9	NRG
DIABLO	NRG
CDKN2A	NRG
TRIM11	NRG
TNFRSF1B	NRG
DNMT1	NRG
ATRX	NRG
STAT3	NRG
MYC	NRG
LEF1	NRG
ID1	NRG

SLC39A7	NRG
FASLG	NRG
MLKL	NRG
CYLD	NRG
CD40	NRG
BCL2L11	NRG
TERT	NRG
AXL	NRG
SIRT1	NRG
PLK1	NRG
ZBP1	NRG
HAT1	NRG
MAPK8	NRG
BNIP3	NRG
SIRT3	NRG
TRAF2	NRG
TARDBP	NRG
HSP90AA1	NRG
DDX58	NRG
PANX1	NRG
FAS	NRG
OTULIN	NRG
MPG	NRG
CFLAR	NRG
MYCN	NRG
TNFRSF1A	NRG
IDH2	NRG
USP22	NRG
FADD	NRG
ITPK1	NRG
