# Supplementary material for: Comprehensive Characterization of Necroptosis-Related lncRNAs in Bladder Cancer Identifies a Novel Signature for Prognosis Prediction
Source: Dis Markers. 2022 Jun 6;2022:2360299. doi: 10.1155/2022/2360299 (PMC9194958; doi:10.1155/2022/2360299)
Supplement: Supplementary 2 — Supplementary Table 2: differentially expressed necroptosis-related lncRNAs. [file 2360299.f2.pdf]

| Gene | logFC | p-value | FDR |
|---|---|---|---|
| AL109955.1 | -1.872 | 0.000 | 0.000 |
| MCF2L-AS1 | 1.148 | 0.048 | 0.049 |
| AC025766.1 | 1.870 | 0.000 | 0.001 |
| AC087286.2 | -1.470 | 0.000 | 0.001 |
| AC073957.3 | 1.298 | 0.001 | 0.001 |
| CDKN2A-DT | 3.485 | 0.005 | 0.008 |
| AL353622.2 | 1.613 | 0.000 | 0.001 |
| AL133467.1 | -1.458 | 0.000 | 0.001 |
| AL161891.1 | 1.240 | 0.000 | 0.000 |
| AC124312.2 | -1.944 | 0.000 | 0.000 |
| AC104785.1 | 1.952 | 0.000 | 0.000 |
| AC074117.1 | 1.253 | 0.000 | 0.000 |
| DNM3OS | -1.015 | 0.000 | 0.000 |
| AC025031.1 | 1.057 | 0.016 | 0.019 |
| AC008764.6 | 1.699 | 0.000 | 0.000 |
| AC023908.3 | 1.875 | 0.000 | 0.000 |
| AC018809.1 | 1.843 | 0.000 | 0.000 |
| AC027449.1 | -3.693 | 0.000 | 0.000 |
| AL158166.1 | 2.809 | 0.000 | 0.000 |
| MIR1-1HG-AS1 | -3.924 | 0.000 | 0.000 |
| AC092803.1 | 5.131 | 0.000 | 0.000 |
| AL357054.4 | -1.214 | 0.000 | 0.000 |
| AC025280.1 | -3.681 | 0.000 | 0.000 |
| TFAP2A-AS1 | 1.771 | 0.000 | 0.000 |
| NCBP2-AS1 | 1.638 | 0.000 | 0.000 |
| AC015802.4 | 1.742 | 0.000 | 0.000 |
| AC027307.3 | -1.366 | 0.000 | 0.000 |
| LINC01322 | 3.325 | 0.011 | 0.015 |
| YEATS2-AS1 | 1.371 | 0.000 | 0.000 |
| MHENCR | 1.017 | 0.000 | 0.000 |
| AC079160.1 | 2.458 | 0.001 | 0.001 |
| AC026740.1 | 1.363 | 0.000 | 0.000 |
| AC087521.1 | -3.231 | 0.000 | 0.000 |
| AC108463.2 | 1.237 | 0.001 | 0.002 |
| AL391807.1 | -1.659 | 0.000 | 0.000 |
| LINC00641 | -1.875 | 0.000 | 0.000 |
| LINC02195 | 2.031 | 0.003 | 0.005 |
| LINC02489 | -3.042 | 0.000 | 0.000 |
| AL023284.4 | 1.282 | 0.002 | 0.003 |
| MIR497HG | -2.119 | 0.000 | 0.000 |
| AC007128.2 | 2.691 | 0.000 | 0.000 |

| | | | |
|---|---|---|---|
| AL133346.1 | -2.413 | 0.000 | 0.000 |
| AC018653.3 | 1.165 | 0.000 | 0.000 |
| AC107959.1 | -1.611 | 0.000 | 0.000 |
| AC036108.3 | -3.975 | 0.000 | 0.000 |
| MCCC1-AS1 | 1.001 | 0.001 | 0.002 |
| AC007622.2 | 1.053 | 0.001 | 0.002 |
| AC008608.2 | 1.067 | 0.000 | 0.000 |
| AL441992.1 | 1.151 | 0.000 | 0.000 |
| TMPO-AS1 | 1.253 | 0.000 | 0.000 |
| AL390067.1 | 1.894 | 0.000 | 0.000 |
| LINC02384 | -1.619 | 0.000 | 0.001 |
| AP005329.1 | 1.112 | 0.000 | 0.000 |
| MIR4435-2HG | 1.324 | 0.000 | 0.000 |
| AL670729.1 | 1.942 | 0.000 | 0.000 |
| AC092376.2 | -2.669 | 0.000 | 0.000 |
| GATA3-AS1 | 1.367 | 0.020 | 0.024 |
| AC005387.1 | 1.570 | 0.000 | 0.000 |
| AP003071.4 | -2.575 | 0.000 | 0.000 |
| AP003086.2 | 1.667 | 0.010 | 0.013 |
| AC139887.1 | 1.122 | 0.002 | 0.003 |
| KDM4A-AS1 | 1.311 | 0.000 | 0.000 |
| WASHC5-AS1 | 1.783 | 0.000 | 0.000 |
| LINC01781 | -2.414 | 0.012 | 0.015 |
| MAGEA10-MAGEA5 | 2.566 | 0.005 | 0.007 |
| LINC00885 | 1.290 | 0.001 | 0.003 |
| XXYLT1-AS2 | -1.574 | 0.000 | 0.000 |
| AL078644.1 | 1.319 | 0.000 | 0.000 |
| U62317.1 | 2.800 | 0.000 | 0.000 |
| AC068790.7 | 1.095 | 0.005 | 0.007 |
| AC053503.4 | -3.147 | 0.000 | 0.000 |
| AL691482.3 | 1.299 | 0.029 | 0.032 |
| LINC00894 | 1.020 | 0.001 | 0.002 |
| MEG9 | -1.966 | 0.000 | 0.000 |
| LINC00941 | 1.955 | 0.031 | 0.033 |
| AL139041.1 | 1.188 | 0.001 | 0.001 |
| AP001625.2 | 1.127 | 0.013 | 0.016 |
| CRTC3-AS1 | 1.112 | 0.000 | 0.000 |
| C8orf44 | 1.097 | 0.000 | 0.000 |
| SH3RF3-AS1 | -1.145 | 0.000 | 0.000 |
| AC090825.1 | -1.750 | 0.000 | 0.000 |
| LINC01081 | -3.557 | 0.000 | 0.000 |
| AL161452.1 | 1.452 | 0.000 | 0.000 |
| ADAMTS9-AS1 | -4.704 | 0.000 | 0.000 |
| AC008735.2 | 1.761 | 0.000 | 0.000 |

| | | | |
|---|---|---|---|
| AC079313.2 | -3.854 | 0.000 | 0.000 |
| AC010618.2 | 1.488 | 0.000 | 0.000 |
| MAGI2-AS3 | -1.924 | 0.000 | 0.000 |
| AL121652.1 | 1.194 | 0.001 | 0.003 |
| AC013553.3 | -1.293 | 0.000 | 0.000 |
| AC244034.2 | 1.127 | 0.011 | 0.015 |
| AC023825.2 | 1.136 | 0.021 | 0.024 |
| ZKSCAN2-DT | 1.319 | 0.000 | 0.000 |
| ZNF252P-AS1 | 2.204 | 0.000 | 0.000 |
| AF001548.1 | -5.007 | 0.000 | 0.000 |
| AC010998.2 | 1.640 | 0.005 | 0.007 |
| MYOSLID | 3.592 | 0.003 | 0.005 |
| NRIR | 2.974 | 0.000 | 0.000 |
| AC015849.4 | 1.386 | 0.005 | 0.008 |
| AL132655.2 | 2.318 | 0.006 | 0.008 |
| ZNF32-AS2 | 1.086 | 0.000 | 0.001 |
| AC007128.1 | 2.650 | 0.000 | 0.000 |
| AC002398.2 | -3.398 | 0.000 | 0.000 |
| AC116407.2 | 1.487 | 0.000 | 0.000 |
| LINC01213 | 1.588 | 0.049 | 0.049 |
| LINC01675 | 1.620 | 0.009 | 0.013 |
| AL157871.5 | 1.356 | 0.001 | 0.001 |
| AC027601.3 | 2.293 | 0.000 | 0.000 |
| AC073046.1 | 1.206 | 0.004 | 0.006 |
| AP003352.1 | 1.063 | 0.000 | 0.000 |
| AC099850.3 | 2.250 | 0.000 | 0.000 |
| AL118511.1 | 1.541 | 0.000 | 0.000 |
| AC010326.3 | 1.242 | 0.000 | 0.000 |
| AL390728.4 | 1.198 | 0.000 | 0.000 |
| AC092910.3 | 1.221 | 0.000 | 0.000 |
| LINC02109 | 3.125 | 0.000 | 0.001 |
| AC010976.2 | -1.948 | 0.000 | 0.000 |
| LINC01411 | 1.896 | 0.016 | 0.019 |
| AC012073.1 | 2.302 | 0.000 | 0.000 |
| AP000892.3 | -3.323 | 0.000 | 0.000 |
| AC092811.1 | -1.769 | 0.000 | 0.000 |
| AL390294.1 | 1.385 | 0.032 | 0.034 |
| AC024361.3 | 1.819 | 0.000 | 0.000 |
| ACTA2-AS1 | -2.719 | 0.000 | 0.000 |
| LINC01355 | 1.491 | 0.000 | 0.000 |
| LINC02577 | 3.101 | 0.000 | 0.000 |
| STAG3L5P-PVRIG2P-PILRB | 1.028 | 0.000 | 0.000 |
| AC004449.1 | 1.931 | 0.000 | 0.000 |

| | | | |
|---|---|---|---|
| PSPC1-AS2 | 1.221 | 0.000 | 0.000 |
| AC018752.1 | -1.878 | 0.001 | 0.002 |
| AC004943.2 | 1.074 | 0.000 | 0.000 |
| SNHG10 | 1.293 | 0.000 | 0.000 |
| CDKN2B-AS1 | 2.806 | 0.002 | 0.003 |
| AC024451.4 | 1.442 | 0.000 | 0.000 |
| NDUFB2-AS1 | 1.181 | 0.000 | 0.000 |
| A2M-AS1 | -1.249 | 0.000 | 0.000 |
| AL928654.2 | 1.038 | 0.001 | 0.001 |
| HAND2-AS1 | -3.973 | 0.000 | 0.000 |
| AL161772.1 | 2.359 | 0.000 | 0.000 |
| AL590729.1 | 1.190 | 0.003 | 0.004 |
| AC010168.2 | 1.555 | 0.000 | 0.000 |
| LINC01820 | 3.737 | 0.002 | 0.003 |
| GHRLOS | 1.567 | 0.000 | 0.000 |
| AC005180.1 | -4.790 | 0.000 | 0.000 |
| AL117329.1 | 3.455 | 0.000 | 0.001 |
| AC091057.1 | 1.788 | 0.000 | 0.000 |
| LINC02100 | 2.565 | 0.000 | 0.000 |
| FLJ12825 | 2.030 | 0.000 | 0.000 |
| AC011477.3 | 1.167 | 0.001 | 0.002 |
| AC027601.1 | 1.252 | 0.000 | 0.000 |
| AC245060.6 | 1.404 | 0.000 | 0.000 |
| AC007637.1 | -1.802 | 0.000 | 0.000 |
| AC011461.1 | 1.372 | 0.000 | 0.000 |
| MORF4L2-AS1 | 1.172 | 0.001 | 0.001 |
| AC010491.1 | 1.656 | 0.000 | 0.000 |
| AC008750.1 | 1.364 | 0.003 | 0.005 |
| AL139352.1 | 1.930 | 0.047 | 0.048 |
| ATP2A1-AS1 | 1.555 | 0.000 | 0.000 |
| AC022893.1 | -2.524 | 0.000 | 0.001 |
| HHIP-AS1 | -2.006 | 0.000 | 0.000 |
| AC006557.1 | 1.444 | 0.001 | 0.002 |
| AL032819.1 | 2.035 | 0.000 | 0.001 |
| C1orf220 | 1.071 | 0.004 | 0.006 |
| GNG12-AS1 | -1.452 | 0.000 | 0.000 |
| AC134043.2 | -1.680 | 0.000 | 0.000 |
| LINC01833 | 2.662 | 0.002 | 0.004 |
| CARMN | -3.721 | 0.000 | 0.000 |
| RUSC1-AS1 | 1.507 | 0.000 | 0.000 |
| MEG3 | -1.183 | 0.000 | 0.000 |
| AC025031.4 | 1.152 | 0.000 | 0.000 |
| MBNL1-AS1 | -3.657 | 0.000 | 0.000 |
| LINC01936 | -1.721 | 0.000 | 0.000 |

| | | | |
|---|---|---|---|
| AC092119.2 | 1.573 | 0.000 | 0.000 |
| ZNF436-AS1 | 1.020 | 0.002 | 0.003 |
| PCAT7 | 1.201 | 0.001 | 0.003 |
| AC105339.2 | 1.270 | 0.000 | 0.000 |
| MAP3K14-AS1 | 1.086 | 0.000 | 0.001 |
| NKILA | 1.922 | 0.009 | 0.012 |
| AP006621.2 | 1.216 | 0.001 | 0.002 |
| AC127024.4 | 1.104 | 0.001 | 0.001 |
| AL590652.1 | 1.075 | 0.002 | 0.003 |
| LINC01342 | 1.247 | 0.046 | 0.047 |
| AC034102.8 | 1.229 | 0.021 | 0.024 |
| AL731567.1 | 1.458 | 0.002 | 0.004 |
| AC093788.1 | 1.403 | 0.000 | 0.000 |
| AC080129.2 | 1.254 | 0.006 | 0.009 |
| GAS6-DT | -1.967 | 0.000 | 0.000 |
| LINC01719 | 1.155 | 0.002 | 0.003 |
| U62317.2 | 1.291 | 0.000 | 0.000 |
| WASIR2 | 2.065 | 0.000 | 0.001 |
| AC010201.2 | 1.181 | 0.001 | 0.002 |
| AL449423.1 | 4.569 | 0.000 | 0.000 |
| AC020928.1 | 2.368 | 0.019 | 0.022 |
| AL161729.4 | 1.697 | 0.000 | 0.000 |
| STAM-AS1 | 2.012 | 0.000 | 0.000 |
| AC124944.3 | 1.443 | 0.000 | 0.000 |
| AC109460.2 | 1.204 | 0.001 | 0.001 |
| AP001189.1 | -1.773 | 0.000 | 0.000 |
| LINC02178 | 1.642 | 0.012 | 0.015 |
| C9orf163 | 2.692 | 0.000 | 0.000 |
| AC106881.1 | -2.457 | 0.000 | 0.000 |
| AC010542.5 | 1.339 | 0.000 | 0.000 |
| LINC01140 | -1.096 | 0.000 | 0.000 |
| TTLL11-IT1 | 2.653 | 0.006 | 0.008 |
| AP000866.5 | 1.601 | 0.005 | 0.007 |
| AC119396.1 | -1.809 | 0.000 | 0.000 |
| AP002761.1 | 2.111 | 0.000 | 0.000 |
| AC005519.1 | 1.230 | 0.000 | 0.000 |
| AP003071.3 | -2.421 | 0.000 | 0.000 |
| LINC01134 | 2.004 | 0.000 | 0.000 |
| BX322562.1 | -1.137 | 0.000 | 0.000 |
| FENDRR | -3.552 | 0.000 | 0.000 |
| CYTOR | 1.313 | 0.002 | 0.004 |
| MIR100HG | -2.702 | 0.000 | 0.000 |
| AC021242.3 | 1.861 | 0.000 | 0.000 |
| GAS8-AS1 | 1.142 | 0.008 | 0.011 |

| | | | |
|---|---|---|---|
| AL021707.8 | 1.058 | 0.001 | 0.002 |
| NARF-IT1 | 1.081 | 0.002 | 0.003 |
| AC020663.2 | 1.190 | 0.002 | 0.004 |
| AC245052.4 | 1.022 | 0.000 | 0.000 |
| AL354993.2 | 1.003 | 0.005 | 0.007 |
| AL445423.1 | -2.429 | 0.000 | 0.000 |
| AC104971.1 | 1.231 | 0.004 | 0.006 |
| AC009148.1 | 2.175 | 0.000 | 0.000 |
| DCST1-AS1 | 2.181 | 0.000 | 0.000 |
| AL117350.1 | 1.141 | 0.042 | 0.043 |
| AL158166.2 | 1.615 | 0.016 | 0.019 |
| AL132642.1 | -3.208 | 0.000 | 0.000 |
| AP001619.1 | 1.302 | 0.011 | 0.014 |
| AL583785.1 | -1.328 | 0.000 | 0.000 |
| AL121832.3 | 2.492 | 0.000 | 0.000 |
| LINC00456 | 2.790 | 0.012 | 0.015 |
| LINC02202 | -2.397 | 0.000 | 0.000 |
| AC012645.2 | 2.034 | 0.003 | 0.005 |
| LINC01410 | 1.537 | 0.000 | 0.000 |
| LINC01082 | -3.900 | 0.000 | 0.000 |
| CAPN10-DT | 1.373 | 0.000 | 0.000 |
| AP001107.5 | -3.921 | 0.000 | 0.000 |
| CEP83-DT | 1.373 | 0.000 | 0.000 |
| AC004253.1 | 1.285 | 0.000 | 0.000 |
| LINC00702 | -3.115 | 0.000 | 0.000 |
| LINC01352 | -2.864 | 0.000 | 0.000 |
| CDC42-IT1 | 1.239 | 0.003 | 0.005 |
| AC016876.3 | 1.661 | 0.000 | 0.000 |
| AC020911.1 | 1.082 | 0.002 | 0.004 |
| AP001189.3 | -1.537 | 0.000 | 0.000 |
| AL157838.1 | 1.543 | 0.000 | 0.000 |
| SNHG9 | 1.108 | 0.002 | 0.004 |
| AL135999.1 | 1.004 | 0.000 | 0.001 |
| AC015819.1 | -1.009 | 0.017 | 0.021 |
| AC004034.1 | 1.972 | 0.000 | 0.000 |
| AC005180.2 | -4.617 | 0.000 | 0.000 |
| PTPRG-AS1 | 1.659 | 0.001 | 0.002 |
| LINC02584 | 1.982 | 0.005 | 0.007 |
| AC008543.3 | 1.235 | 0.029 | 0.032 |
| LINC02104 | -1.300 | 0.000 | 0.001 |
| AC021491.2 | 1.273 | 0.004 | 0.006 |
| AL592211.1 | 1.009 | 0.036 | 0.038 |
| AC083906.3 | 2.762 | 0.015 | 0.019 |
| MCM3AP-AS1 | 1.015 | 0.000 | 0.000 |

| | | | |
|---|---|---|---|
| AC084125.2 | 1.612 | 0.000 | 0.000 |
| AC021683.1 | -1.951 | 0.000 | 0.000 |
| Z94721.1 | 1.829 | 0.000 | 0.000 |
| AC009299.2 | -2.088 | 0.000 | 0.000 |
| AC010271.2 | 1.067 | 0.042 | 0.043 |
| TSPOAP1-AS1 | -1.357 | 0.000 | 0.000 |
| NARF-AS1 | 3.303 | 0.000 | 0.000 |
| AC124312.3 | -1.560 | 0.000 | 0.000 |
| AC078795.1 | 1.627 | 0.000 | 0.000 |
| AC108860.2 | 1.839 | 0.000 | 0.000 |
| AL138995.1 | -1.532 | 0.000 | 0.000 |
| AC013403.2 | -1.103 | 0.000 | 0.000 |
| AC127070.1 | 1.197 | 0.003 | 0.005 |
| AL109741.1 | -1.341 | 0.000 | 0.000 |
| AL445490.1 | 2.154 | 0.005 | 0.007 |
| ZNF32-AS1 | 1.417 | 0.000 | 0.000 |
| AP001628.1 | 1.327 | 0.000 | 0.001 |
| AC132192.2 | 1.118 | 0.000 | 0.000 |
