# Supplementary material for: Comprehensive Characterization of Necroptosis-Related lncRNAs in Bladder Cancer Identifies a Novel Signature for Prognosis Prediction
Source: Dis Markers. 2022 Jun 6;2022:2360299. doi: 10.1155/2022/2360299 (PMC9194958; doi:10.1155/2022/2360299)
Supplement: Supplementary 3 — Supplementary Table 3: 42 differentially expressed necroptosis-related lncRNAs were identified by univariate Cox analysis. [file 2360299.f3.pdf]

Gene	HR	HR.95L	HR.95H	p-value
AC104785.1	0.595	0.421	0.842	0.003
AC074117.1	0.832	0.732	0.946	0.005
AC018809.1	0.614	0.462	0.817	0.001
AC015802.4	0.223	0.089	0.557	0.001
AL391807.1	1.331	1.171	1.513	0.000
AC018653.3	0.768	0.641	0.921	0.004
AP005329.1	0.439	0.249	0.773	0.004
AC005387.1	0.654	0.493	0.868	0.003
AL078644.1	1.676	1.159	2.424	0.006
AC068790.7	0.249	0.095	0.651	0.005
C8orf44	0.727	0.604	0.876	0.001
AC010618.2	0.592	0.408	0.859	0.006
AC023825.2	0.162	0.042	0.619	0.008
ZKSCAN2-DT	0.676	0.518	0.882	0.004
AL132655.2	0.573	0.379	0.866	0.008
ZNF32-AS2	0.684	0.528	0.885	0.004
AP003352.1	0.877	0.807	0.954	0.002
AC010326.3	0.938	0.896	0.983	0.007
LINC01355	0.815	0.703	0.944	0.007
STAG3L5P-PVRIG2P-PILRB	0.540	0.398	0.734	0.000
AC024451.4	0.414	0.237	0.723	0.002
AC010168.2	0.712	0.575	0.882	0.002
FLJ12825	0.187	0.059	0.593	0.004
AC011477.3	0.885	0.808	0.968	0.008
AC010491.1	0.289	0.133	0.627	0.002
LINC01833	0.892	0.823	0.966	0.005
LINC01936	4.588	1.576	13.356	0.005
ZNF436-AS1	0.859	0.773	0.956	0.005
MAP3K14-AS1	0.671	0.510	0.884	0.004
AL731567.1	0.919	0.870	0.971	0.003
AC093788.1	0.606	0.461	0.796	0.000
AC080129.2	0.672	0.508	0.887	0.005
AC010201.2	0.545	0.354	0.839	0.006
AC010542.5	0.840	0.760	0.929	0.001
BX322562.1	1.224	1.060	1.414	0.006
AL021707.8	0.815	0.735	0.904	0.000
AL583785.1	1.067	1.020	1.116	0.005
AC004253.1	0.713	0.554	0.917	0.008
AC020911.1	0.162	0.053	0.496	0.001
AC008543.3	0.342	0.164	0.711	0.004

AC009299.2	1.606	1.164	2.215	0.004
ZNF32-AS1	0.644	0.472	0.879	0.006
