# Supplementary material for: Comprehensive Characterization of Necroptosis-Related lncRNAs in Bladder Cancer Identifies a Novel Signature for Prognosis Prediction
Source: Dis Markers. 2022 Jun 6;2022:2360299. doi: 10.1155/2022/2360299 (PMC9194958; doi:10.1155/2022/2360299)
Supplement: Supplementary 4 — Supplementary Table 4: 12 prognosis-related differentially expressed necroptosis-related lncRNAs. [file 2360299.f4.pdf]

Gene	Coef
AC015802.4	-0.488
AL391807.1	0.166
AL078644.1	0.204
AC023825.2	-1.212
AL132655.2	-0.005
AP003352.1	-0.045
STAG3L5P-PVRIG2P-PILRB	-0.172
AC024451.4	-0.084
MAP3K14-AS1	-0.140
AL731567.1	-0.025
AC010542.5	-0.003
AC009299.2	0.123
