# Supplementary material for: Comprehensive Characterization of Necroptosis-Related lncRNAs in Bladder Cancer Identifies a Novel Signature for Prognosis Prediction
Source: Dis Markers. 2022 Jun 6;2022:2360299. doi: 10.1155/2022/2360299 (PMC9194958; doi:10.1155/2022/2360299)
Supplement: Supplementary 5 — Supplementary Table 5: univariate and multivariate Cox regression analyses for the NerRLsig as an independent prognostic factor. [file 2360299.f5.pdf]

Variables	univariate Cox regression				multivariate Cox regression			
	HR	HR.95L	HR.95H	p-value	HR	HR.95L	HR.95H	p-value
Age	1.034	1.018	1.051	0.000	1.030	1.014	1.046	0.000
Gender	0.894	0.644	1.241	0.504				
Stage	1.743	1.437	2.114	0.000	1.634	1.342	1.989	0.000
Grade	0.347	0.086	1.403	0.138				
riskScore	2.262	1.664	3.076	0.000	2.058	1.574	2.692	0.000
