# Supplementary material for: Comprehensive Characterization of Necroptosis-Related lncRNAs in Bladder Cancer Identifies a Novel Signature for Prognosis Prediction
Source: Dis Markers. 2022 Jun 6;2022:2360299. doi: 10.1155/2022/2360299 (PMC9194958; doi:10.1155/2022/2360299)
Supplement: Supplementary 6 — Supplementary Table 6: the risk score correlated with clinicopathological features in bladder cancer. [file 2360299.f6.pdf]

| Covariates | subgroups | Total | high | low | chi | p-value |
|---|---|---|---|---|---|---|
| Age | <=65 | 160(39.41%) | 76(37.07%) | 84(41.79%) | 0.759 | 0.384 |
| | >65 | 246(60.59%) | 129(62.93%) | 117(58.21%) | | |
| Gender | FEMALE | 107(26.35%) | 63(30.73%) | 44(21.89%) | 3.645 | 0.056 |
| | MALE | 299(73.65%) | 142(69.27%) | 157(78.11%) | | |
| T | T1-2 | 201(49.51%) | 88(42.93%) | 113(56.22%) | 8.567 | 0.014 |
| | T3-4 | 199(49.01%) | 115(56.1%) | 84(41.79%) | | |
| | Unknown | 6(1.48%) | 2(0.98%) | 4(1.99%) | | |
| N | N0 | 236(58.13%) | 115(56.1%) | 121(60.2%) | 5.620 | 0.060 |
| | N1-3 | 128(31.53%) | 74(36.1%) | 54(26.87%) | | |
| | Unknown | 42(10.34%) | 16(7.8%) | 26(12.94%) | | |
| M | M0 | 195(48.03%) | 88(42.93%) | 107(53.23%) | 4.631 | 0.099 |
| | M1 | 11(2.71%) | 7(3.41%) | 4(1.99%) | | |
| | Unknown | 200(49.26%) | 110(53.66%) | 90(44.78%) | | |
| Stage | Stage I-II | 131(32.27%) | 53(25.85%) | 78(38.81%) | 10.253 | 0.006 |
| | Stage III-IV | 273(67.24%) | 152(74.15%) | 121(60.2%) | | |
| | Unknown | 2(0.49%) | 0(0%) | 2(1%) | | |
| Grade | High Grade | 383(94.33%) | 198(96.59%) | 185(92.04%) | 3.936 | 0.140 |
| | Low Grade | 20(4.93%) | 6(2.93%) | 14(6.97%) | | |
| | Unknown | 3(0.74%) | 1(0.49%) | 2(1%) | | |
