# Supplementary material for: Comprehensive Characterization of Necroptosis-Related lncRNAs in Bladder Cancer Identifies a Novel Signature for Prognosis Prediction
Source: Dis Markers. 2022 Jun 6;2022:2360299. doi: 10.1155/2022/2360299 (PMC9194958; doi:10.1155/2022/2360299)
Supplement: Supplementary 7 — Supplementary Table 7: gene set enrichment analysis (GSEA) of high-risk group based on the necroptosis-related lncRNA signature (NerRLsig). [file 2360299.f7.pdf]

NAME	NES	NOM p-val	FDR q-val
KEGG_FOCAL_ADHESION	2.309	0.000	0.000
KEGG_ARRHYTHMOGENIC_RIGHT_VENTRICULAR_CARDIOMYOPATHY_ARVC	2.271	0.000	0.000
KEGG_MELANOMA	2.215	0.000	0.000
KEGG_PATHOGENIC_ESCHERICHIA_COLI_INFECTION	2.225	0.000	0.000
KEGG_REGULATION_OF_ACTIN_CYTOSKELETON	2.239	0.000	0.000
KEGG_ECM_RECEPTOR_INTERACTION	2.261	0.000	0.000
KEGG_HYPERTROPHIC_CARDIOMYOPATHY_HCM	2.117	0.000	0.002
KEGG_DILATED_CARDIOMYOPATHY	2.083	0.000	0.003
KEGG_AXON_GUIDANCE	2.058	0.000	0.004
KEGG_PRION_DISEASES	2.005	0.000	0.009
KEGG_PATHWAYS_IN_CANCER	1.972	0.000	0.011
KEGG_WNT_SIGNALING_PATHWAY	1.893	0.000	0.018
KEGG_LEUKOCYTE_TRANSENDOTHELIAL_MIGRATION	1.901	0.000	0.018
KEGG_CALCIUM_SIGNALING_PATHWAY	1.906	0.000	0.018
KEGG_GAP_JUNCTION	1.908	0.002	0.019
KEGG_BLADDER_CANCER	1.914	0.002	0.020
KEGG_GLIOMA	1.917	0.000	0.021
KEGG_RENIN_ANGIOTENSIN_SYSTEM	1.868	0.000	0.022
KEGG_TGF_BETA_SIGNALING_PATHWAY	1.864	0.008	0.022
KEGG_GLYCOSAMINOGLYCAN_BIOSYNTHESIS_CHONDROITIN_SULFATE	1.851	0.013	0.024
KEGG_SMALL_CELL_LUNG_CANCER	1.784	0.004	0.036
KEGG_PROTEASOME	1.788	0.017	0.036
KEGG_PANCREATIC_CANCER	1.779	0.004	0.036

KEGG_ADHERENS_JUNCTION	1.797	0.006	0.036
KEGG_CYTOKINE_CYTOKINE_RECEPTOR_INTERACTION	1.793	0.006	0.036
KEGG_TIGHT_JUNCTION	1.769	0.006	0.037
KEGG_VIRAL_MYOCARDITIS	1.772	0.014	0.037
KEGG_MAPK_SIGNALING_PATHWAY	1.798	0.002	0.038
KEGG_RENAL_CELL_CARCINOMA	1.747	0.012	0.044
