# Supplementary material for: Comprehensive Characterization of Necroptosis-Related lncRNAs in Bladder Cancer Identifies a Novel Signature for Prognosis Prediction
Source: Dis Markers. 2022 Jun 6;2022:2360299. doi: 10.1155/2022/2360299 (PMC9194958; doi:10.1155/2022/2360299)
Supplement: Supplementary 8 — Supplementary Table 8: the relationship between MAP3K14-AS1 and STAG3L5P-PVRIG2P-PILRB with drug sensitivity. [file 2360299.f8.pdf]

Gene	Drug	cor	p-value
MAP3K14-AS1	Ibrutinib	0.387	0.002
	Lapatinib	0.367	0.004
	Selumetinib	-0.366	0.004
	Everolimus	0.354	0.006
	Dacomitinib	0.337	0.008
	Temsirolimus	0.333	0.009
	Gefitinib	0.329	0.010
	ARRY-162	-0.306	0.018
	Neratinib	0.305	0.018
	Afatinib	0.303	0.019
	Encorafenib	-0.302	0.019
	Pipamperone	-0.280	0.030
	Erlotinib	0.276	0.033
	Cobimetinib (isomer 1)	-0.274	0.034
	Vemurafenib	-0.272	0.035
	Carmustine	-0.268	0.039
STAG3L5P-PVRIG2P-PILRB	DECITABINE	0.264	0.041
